# Torque teno viruses exhaust and imprint the human immune system via the HLA-E/NKG2A axis

**DOI:** 10.3389/fimmu.2024.1447980

**Published:** 2024-09-04

**Authors:** Hannes Vietzen, Cara Simonitsch, Benjamin Friedel, Sarah M. Berger, Laura M. Kühner, Philippe L. Furlano, David M. Florian, Irene Görzer, Maximilian Koblischke, Judith H. Aberle, Elisabeth Puchhammer-Stöckl

**Affiliations:** Center for Virology, Medical University Vienna, Vienna, Austria

**Keywords:** TTV, Torque teno virus, NKG2A, HLA-E, immune evasion, immune regulation

## Abstract

The ubiquitous Torque teno virus (TTV) establishes a chronically persistent infection in the human host. TTV has not been associated with any apparent disease, but, as part of the human virome, it may confer a regulatory imprint on the human immune system with as yet unclear consequences. However, so far, only few studies have characterized the TTV-specific immune responses or the overall immunological imprints by TTV. Here, we reveal that TTV infection leads to a highly exhausted TTV-specific CD8^+^ T-cell response, hallmarked by decreased IFN-γ production and the expression of the inhibitory NKG2A-receptor. On a functional level, we identified a panel of highly polymorphic TTV-encoded peptides that lead to an expansion of regulatory NKG2A^+^ natural killer, NKG2A^+^CD4^+^, and NKG2A^+^CD8^+^ T cells via the stabilization of the non-classical HLA-E molecule. Our results thus demonstrate that TTV leads to a distinct imprint on the human immune system that may further regulate overall human immune responses in infectious, autoimmune, and malignant diseases.

## Introduction

Viruses that cause life-long chronically persistent or latent infections constitute a substantial part of the human virome (1). Torque teno virus (TTV) is a ubiquitous negative-strand DNA virus, which belongs to the *Anelloviridae* family, *Alphatorquevirus* genus, and has a circular genome of ~3.9 kbp that encodes for four open reading frames (ORFs) (2). TTV is an exceptionally polymorphic virus, and numerous strains have been identified so far, which are currently grouped into 20 TTV species (2, 3). TTV is omnipresent in the human population and the most abundant eukaryotic virus of the human virome (1). So far, TTV has not been associated with any apparent disease, and it is thus hypothesized that it may contribute to a personal virus flora that shapes the human immune system and thus may positively influence human physiology (4). After primary infection, TTV establishes a probably lifelong chronically persistent infection in the host, and, as a result, TTV-DNA is detectable in various body compartments in a substantial part of humans (5). So far, only few studies have characterized the TTV-specific immune responses (6, 7). It is still unclear, which mechanism enables TTV to evade the TTV-specific immune responses of the host and which imprints TTV may have on the human immune system. However, increased TTV-DNA levels in immunosuppressed patients with severely impaired adaptive immune responses suggest that CD8^+^ T cells play a key role in limiting TTV (7–11).

Moreover, the human cytomegalovirus (HCMV), a herpesvirus, infects large parts of the population worldwide (12). In contrast to TTV, however, HCMV causes latent infections after primary infection from which only sporadically reactivations are observed. This is mostly due to HCMV replication being mainly controlled by human CD8^+^ T-cell responses (13). HCMV evolved, however, a multitude of immune evasive mechanisms. The HCMV-encoded UL40-derived peptide stabilizes the non-classical MHC class I molecule HLA-E on the surface of HCMV-infected cells. By this mechanism, HCMV leads to the stimulation of the inhibitory NKG2A receptor, which is expressed on distinct natural killer (NK) and T-cell subsets (14). The HCMV UL40-mediated stimulation of the NKG2A-receptor results consequently in the inhibition and the proliferation of NKG2A-expressing NK and T cells (15).

The aim of the present study was to analyze and characterize the human CD8^+^ T-cell responses against TTV. Furthermore, we aimed to evaluate whether TTV, similar to HCMV, utilizes the HLA-E/NKG2A axis for potent immune evasion. Finally, we aimed to assess to which extent TTV infections may imprint the human immune system.

## Methods

### Ethics statement

The study was approved by the institutional review board of the Medical University of Vienna. Written informed consent was obtained from all study participants.

### Cells

The mouse lymphoma cell line RMA-S/HLA-E/LFA-3 was kindly provided by Chiara Romagnani (DRFZ, Berlin) and propagated in RPMI-1640 + 20 mM glutamine + 10% FCS + 20 μM β-mercaptoethanol + 100 U/ml penicillin-streptomycin (all from Thermo Fisher, Waltham, MA, USA) + 400 μg/ml hygromycin B + 1 mg/ml G418 (both from Invitrogen, Waltham, MA, USA). K562–HLA-E*0103/0103 cells were kindly provided by Thorbald van Hall (Leiden University Medical Center) and cultured in IMDM + 10% FCS. PBMCs from healthy blood donors (Austrian Red Cross) were isolated from whole blood by density gradient centrifugation using Ficoll-Paque Plus (GE Healthcare).

### Study participants

In total, 124 healthy HCMV-seropositive and TTV-DNA-positive blood donors (BD001-124) and 120 healthy TTV-DNA-positive and HCMV-seronegative healthy blood donors (BD125-BD249) were included in this study. For BD004-249, seven additional plasma samples were available in 6-month intervals (± 3.5 weeks).

### TTV-PCR and genotyping

Viral DNA was isolated from plasma samples using the TanBead Nucleic Acids Extraction Kit (Taiwan Advanced Nanotech Inc.). Nucleic acids were eluted in 50 μl nuclease-free H_2_O. TTV-DNA was detected and quantified by TaqMan real-time PCR covering the conserved region common to all currently known TTV strains, as described previously (8, 16). All blood donors had a viral load of >7.5 × 10^4^ copies/ml in the first plasma sample. TTV-positive samples were genotyped by TTV genotype PCR (17, 18), followed by Sanger sequencing. For the determination of the TTV species, the sequences were subjected to alignment and phylogenetic analyses including BLAST (Basic Local Alignment Search Tool) (19) and UPGMA (unweighted pair group method with arithmetic mean). The blood donor BB001 was selected as a reference for all predictions, due to a high viral load and a single infection with well characterized TTV strain TTV-BNI-700611 (GenBank: MH017571.1, *Alphatorquevirus* homin1). From BB001, PBMCs and an additional plasma sample for TTV sequencing were available from the same time point.

### HCMV serology

HCMV-specific IgG antibodies were detected and quantified by ELISA (Euroimmune, Lübeck, Germany).

### HLA-I allele genotyping

HLA-I alleles of blood donor BD001 (A*02:01, A*32:01, B*13:02, B*40:303, C*02:02, and C*06:02) were determined by HLA genotyping, as described previously (20). HLA-E genotyping was performed using a recently published TaqMan assay and HLA-E*0101- and HLA-E*0103-specific probes (21, 22). BD001 was confirmed to encode for the homozygous HLA-E*0103/0103 allelic variant.

### Next-generation sequencing

The viral metagenome was assessed in the first plasma sample using the Illumina NextSeq Platform as recently described in detail (23) and revealed in all blood donors > 114,000 high-quality TTV-specific reads. TTV-specific reads were assembled into TTV-specific contiguous sequences (contigs) using 49,087 viral complete genomes obtained from the NCBI GenBank nuccore database (23). The number of functional-inhibitory peptides was then assessed by two independent methods: first, TTV ORF1-specific contiguous sequences were mapped to published TTV sequences, shown in **Supplementary Table S2**, using MUSCLE (**MU**ltiple Sequence Comparison by Log- Expectation) Sequence Alignment (EMBL’s European Bioinformatics Institute) (24). Here, we used the ICTV threshold of 69% nucleotide identity of the complete ORF1 (2) to annotate the corresponding TTV strains. The number of inhibitory peptides was then assessed in the published TTV strains and reassigned to the TTV strains, found in the blood donors. Second, TTV-specific contiguous sequences were directly six-frame translated using EMBOSS SIXPACK and then blasted against all known inhibitory peptides, shown in **Supplementary Table S2**, using MUSCLE. In all included blood donors, both methods revealed an identical number of inhibitory peptides.

### Peptide-MHC I binding predictions

Predictions were performed using the Consensus Tool “IEDB Recommended 2020.09” of the immune epitope database analysis resource IEDB (25) for the sequences of all four ORFs of TTV-BNI-700611-G4-CSF (accession number MH017571), accessed via Uniprot, and five of the six HLA-I alleles of blood donor BB001 (A*02:01, A*32:01, B*13:02, C*02:02, and C*06:02). HLA-B*40:303 was not available on IEDB. In addition, the top 1% HLA-C peptides, not already covered by HLA-A/B alleles, were chosen. HLA-E peptides were predicted using the HLA-E*0103 allele. For all these predictions, a peptide length of nine amino acids was chosen. All peptides that had a percentile rank of <3% for at least one HLA-I allele were defined as strong binders and were selected for peptide synthesis.

### IFNγ ELISPOT assay

Peripheral blood mononuclear cells (PBMCs) were isolated from whole blood by density gradient centrifugation using Ficoll-Paque Plus (GE Healthcare) according to the manufacturer’s instructions. After isolation, PBMCs were cryopreserved in FCS (Capricorn) containing 7.5% DMSO (Thermo Scientific) in liquid nitrogen for future use.

Frozen PBMCs were thawed and diluted 1:10 in RPMI-1640 medium (Sigma-Aldrich) containing CTL Wash Supplement (Cellular Technology Limited), 1% glutamine (Sigma-Aldrich), and 1,000 U of a Pierce nuclease (250 U/μl, Thermo Scientific). PBMCs were depleted of CD4^+^ T cells using anti-CD4 antibody-coupled immunomagnetic beads and magnetic columns (Miltenyi Biotec), according to the company’s instructions. CD4-depleted PBMCs were resuspended in serum-free AIM-V medium (Gibco) and incubated overnight at 37°C and 5% CO_2_. Cell viability and depletion efficiency of depleted PBMCs were monitored using flow cytometry analysis, as described below. Depleted PBMCs contained <20% dead cells and <1% CD4^+^ T cells.

A total of 124 predicted TTV peptides of all four open reading frames were purchased at JPT (**Supplementary Table S1**, JPT Peptide Technologies). The purity of all peptides was >70% as determined via high-performance liquid chromatography HPLC. Peptides were arranged into four maxi-pools with each pool containing all the peptides of the according open reading frames (ORF1–ORF4). ORF1 contains 89 peptides, ORF2 8 peptides, ORF3 10 peptides, and ORF4 17 peptides. Peptide pool ORF1 was additionally divided into eight subpools (SP1–SP8) containing 9–14 single peptides to facilitate the identification of T-cell epitopes. Lyophilized peptides were dissolved in DMSO and diluted in AIM-V medium (both from Thermo Fisher).

For non-pre-expanded CD8^+^ T cell assays, CD4-depleted PBMCs were cultured in filtered RPMI-1640 medium containing 10% heat-inactivated FCS, 0.5% PenStrep 50 IU/ml, 1% L-glutamine 2mM, 1% Hepes buffer 10 mM (all from Sigma-Aldrich), and 50 IU/ml IL-2 (Peprotech) with peptide pools (final peptide concentration of 1 µg/ml) for 24 h, before testing them in IFNγ ELISPOT assays.

For pre-expanded CD8^+^ T cell assays, CD4-depleted PBMCs were cultured in filtered RPMI-1640 medium containing 10% heat-inactivated FCS, 0.5% PenStrep 50 IU/ml, 1% L-glutamine 2mM, 1% Hepes buffer 10 mM, and 50 IU/ml IL-2 and with respective peptide pools (final peptide concentration of 1 µg/ml) for 11 days to allow expansion of peptide-specific memory CD8^+^ T cells, before testing them in IFNγ ELISPOT assays. On day 5, 50% of the medium was exchanged with medium containing 100 IU/ml IL-2. To test subpools and single peptides, CD4-depleted PBMCs not used in the ELISPOT assays were further expanded using a long-term expansion medium (RPMI-1640 with 30% FCS, 1% Glutamax (Gibco), 1% sodium pyruvate (Gibco), 1% non-essential amino acids (Sigma-Aldrich), 1% Hepes, 0.5% PenStrep, and 50 IU/ml IL-2 with supplementary CD3/CD28 dyna-beads (Invitrogen) for the stimulation of T-cell proliferation. Expansion with Dyna beads was necessary since the PBMCs of the blood donors were limited. CD4-depleted PBMCs were incubated at 37°C and 5% CO_2_, split every 2–3 days, and fed at least once a week with 4 × 10^4^ beads per well. When the T-cell number was sufficient, they were harvested, and beads were removed according to the instructions of the company using the DynaMag-15 magnet (Invitrogen). After resting the expanded PBMCs overnight in serum-free AIM-V medium, they were tested in ELISpot assays. All experiments were performed in parallel with an HCMV-derived peptide pool, which contains 28 MHC class I and 14 MHC II-restricted peptides (Mabtech).

For IFNγ ELISPOT assays, plates (Merck-Millipore) were coated with 1.5 µg mouse-anti-human IFNγ (1-D1K, Mabtech). Blocking was performed using a 5% BSA/PBS solution. CD4-depleted PBMCs (2 × 10^5^/per well) were incubated at 37°C and 5% CO_2_ for 48 h in a humidified incubator with peptides with a final peptide concentration of 2 µg/ml, AIM-V medium (Gibco) as a negative control or phytohemagglutinin (PHA-Sigma) with a final concentration of 0.5 µg/ml as a positive control. After incubation and washing, spots were developed with 0.1 µg biotin-conjugated mouse-anti-human IFNγ (7-B6-1, Mabtech), streptavidin-coupled alkaline phosphatase (ALP, 1:1,000, 3310-10, Mabtech), and 5-bromo-4-chloro-3-indolylphosphate/nitroblue tetrazolium (BCIP/NBT, B5655, Sigma). Plates were analyzed using the Bioreader 5000 with the Bioreader Software 10 (BIOSYS). Data were computed as spot-forming cells (SFCs) per 1 × 10^6^ CD4-depleted PBMCs. T-Cell responses were considered positive when peptide stimulation was above the mean+3SD of the negative control of all tests (48 SFCs/1 × 10^6^ CD4-depleted PBMCs). The response to a single peptide was defined as positive if also the corresponding ORF pool and sub-pool were positive in at least three independent experiments.

### Tetramer assays

PBMCs were isolated from whole blood by density gradient centrifugation using Ficoll-Paque Plus. After isolation, PBMCs were cryopreserved in FCS containing 7.5% DMSO in liquid nitrogen for future use. CD8^+^ T cells were enriched by magnetic labeling using a human CD8^+^ T-cell Isolation Kit according to the manufacturer’s instructions (Miltenyi Biotec). Cell viability and depletion efficiency of depleted PBMCs were monitored using flow cytometry analysis, as described below. CD8^+^ T cells contained <20% dead cells and >95% CD8^+^ T cells. Individual or pooled APC-conjugated HLA-A*02:01, HLA-A*03:01, HLA-A*11:01, and HLA-A*24:02 tetramers (Tetramer Shop) were loaded with 8 µg/ml of individual or pooled TTV-encoded LEYHGGLYS, IEGLPLWAA, KHTYRPYLF, CDLPLLTIF, and TLFHQKEVL peptides or a HCMV-derived peptide pool, respectively, according to the manufacturer’s instruction. Loaded tetramer pools were incubated together in 1 ml Opti-MEM (Thermo Fisher) with non-pre-expanded 2.5 × 10^6^ CD8^+^ T cells at 37°C and 5% CO_2_ for 7 h. After 1 h, brefeldin A (5.0 µg/ml, BioLegend) was added. TTV- or HCMV-specific and activated (IFNγ) cells were assessed by flow cytometry as described below. For some experiments, APC-tetramer-conjugated TTV-specific CD8^+^ T cells were sorted using FACSAria Fusion (BD Bioscience, Franklin Lakes, NJ, USA). Sorted cells were re-stained with pooled PE-conjugated HLA-A*02:01, HLA-A*03:01, HLA-A*11:01, and HLA-A*24:02 tetramers (Tetramer Shop), loaded with 8 µg/ml of pooled TTV-encoded LEYHGGLYS, IEGLPLWAA, KHTYRPYLF, CDLPLLTIF, and TLFHQKEVL peptides or random nine amino acid peptides (negative control, GenScript). Cells were incubated at 37°C and 5% CO_2_ for 7 h and analyzed by flow cytometry as described below.

### HLA-upregulation assay

HLA-E surface stabilization assays were performed as previously described (26, 27). In brief, the mouse lymphoma cell line RMA-S/HLA-E/LFA-3 was maintained in RPMI-1640 + 20 mM glutamine + 10% FCS + 20 μM β-mercaptoethanol + 100 U/ml penicillin-streptomycin (all Thermo Fisher) + 400 μg/ml hygromycin B + 1 mg/ml G418 (both from Invitrogen). A total of 5×10^5^ cells/ml) were incubated together with 300 μM of the HCMV-encoded *VMAPRTLIL* or indicated TTV-derived peptides (Peptides & Elephants) in 1 ml Opti-MEM for 16 h at 37°C. Peptide-pulsed cells were either fixed and stained for HLA-E surface expression by flow cytometry analysis or washed with Opti-MEM and used for NKG2A proliferation assays.

### NKG2A proliferation assays

PBMCs were isolated from whole blood by density gradient centrifugation using Ficoll-Paque Plus. After isolation, PBMCs were cryopreserved in FCS containing 7.5% DMSO in liquid nitrogen for future use. CD8^+^ T cells, CD4^+^ T cells, or CD56^+^ NK cells were enriched by magnetic labeling using a human CD8^+^ T-cell Isolation Kit, CD4^+^ T-cell Isolation Kit, or CD56^+^ NK cell Isolation Kit, respectively, according to the manufacturer’s instructions (Miltenyi Biotec). Cell viability and depletion efficiency of depleted PBMCs were monitored using flow cytometry analysis, as described below. Peptide pulsed RMA-S/HLA-E/LFA-3 cells were inactivated using 20 µg/ml Mitomycin C (Sigma Aldrich) at 37 C for 30 min. The cells were then co-cultivated in 1 mL RPMI-1640 + 20 mM glutamine + 10% FCS with CFSE (CellTrace CFSE Cell Proliferation Kit, Thermo Fisher) stained MACS-enriched CD8^+^ T cells, CD4^+^ T cells, or CD56^+^ NK cells for 7 days (E:T, 2:1). Each 3 days, fresh peptide-pulsed and inactivated pulsed RMA-S/HLA-E/LFA cells were added to the culture. Cells were then harvested, fixed with the FIX & PERM Cell Fixation & Cell Permeabilization Kit (Thermo Scientific), and analyzed for CFSE and the expression of NKG2A by flow cytometry, as described below. For each experiment and each blood donor, additional α-NKG2A blocking monoclonal antibody controls (10 μg/ml, Monalizumab, Innate Pharma) and non-peptides controls were included.

### NKG2A-inhibition assays

PBMCs were isolated from whole blood by density gradient centrifugation using Ficoll-Paque Plus. After isolation, PBMCs were cryopreserved in FCS containing 7.5% DMSO in liquid nitrogen for future use. CD8^+^ T cells, CD4^+^ T cells, or CD56^+^ NK cells were enriched by magnetic labeling using a human CD8^+^ T-cell Isolation Kit, CD4^+^ T-cell Isolation Kit, or CD56^+^ NK cell Isolation Kit, respectively, according to the manufacturer’s instructions (Miltenyi Biotec). Cell viability and depletion efficiency of depleted PBMCs were monitored using flow cytometry analysis, as described below. TAP-competent K562–HLA-E*0103/0103 cells were cultured in IMDM + 10% FCS. A total of 5 × 10^5^ cells/ml were incubated together with 300μM of the HCMV-encoded *VMAPRTLIL* or indicated TTV-derived peptides (Peptides & Elephants) in 1 ml Opti-MEM for 16 h at 37°C. Total MACS-enriched CD8^+^ T cells, CD4^+^ T cells, or CD56^+^ NK cells were pre-activated overnight in RPMI, 10% FCS, 1% L-glutamine, and 100 U/ml IL-2 (PeproTec). Cells were then harvested by centrifugation at 400 × g for 5 min and washed once with Opti-MEM I Reduced Serum Medium. The cells were then cultured together with peptide-pulsed K562–HLA-E*0103/0103 cells (E:T, 1:2) and 5 µL mouse anti-human CD107a-APC-H7 (BD) at 37°C and 5% CO_2_ for 7 h in 1 ml Opti-MEM. After 1 h, brefeldin A (5.0 µg/ml, Biolegend) was added. Cells were then harvested, fixed with the FIX & PERM Cell Fixation & Cell Permeabilization Kit and analyzed for the expression of NKG2A, CD107a, and IFNγ by flow cytometry, as described below. For each experiment and each blood donor, additional α-NKG2A blocking monoclonal antibody controls (10 μg/ml, Monalizumab, Innate Pharma) and non-peptides controls were included.

### Flow cytometry

The following conjugated mouse anti-human mAb were used for flow cytometry: BV510-CD3 (clone UCHT1), BV421-CD4 (clone OKT4), BV421-CD8 (clone RPA-T8), BV421-CD56 (clone NCAM16.2), PE/Cy7-IFNγ (clone B27), APC/H7-CD107a (clone H4A3), PerCPCy5.5-PD-1 (clone EH12.1), BV510-NKG2A (clone 131411), BV510-NKG2C (clone 134591), and PE-LAG-3 (clone T47-530) (all from BD), PE/Cyanine7–HLA-E (3D12), PE/Cy7-TIGIT (clone A15153G), and APC/Cy7-TIM-3 (clone F38-2E2) (all from BioLegend). Dead cells were identified using LIVE/DEAD Fixable Green Dead Cell Stain Kit or 7-Aminoactinomycin D (7-AAD, Invitrogen). Flow cytometry analysis was performed on a FACSCanto2 platform and FACSDiva Version 10.7.2 (BD).

### Statistical analysis

Outliers in the functional ELISpot and flow cytometry assays were first determined and excluded using the Grubbs’ test, and statistical differences were then assessed with the Mann–Whitney test, the Kruskal–Wallis test, or two-way ANOVA. Associations between the variables were assessed by simple linear regression. p-Values < 0.05 were considered statistically significant. All statistical analyses were conducted using GraphPad Prism 10 (GraphPad Software).

## Results

### Characterization of TTV-specific CD8^+^ T-cell responses

**Figure 1:**
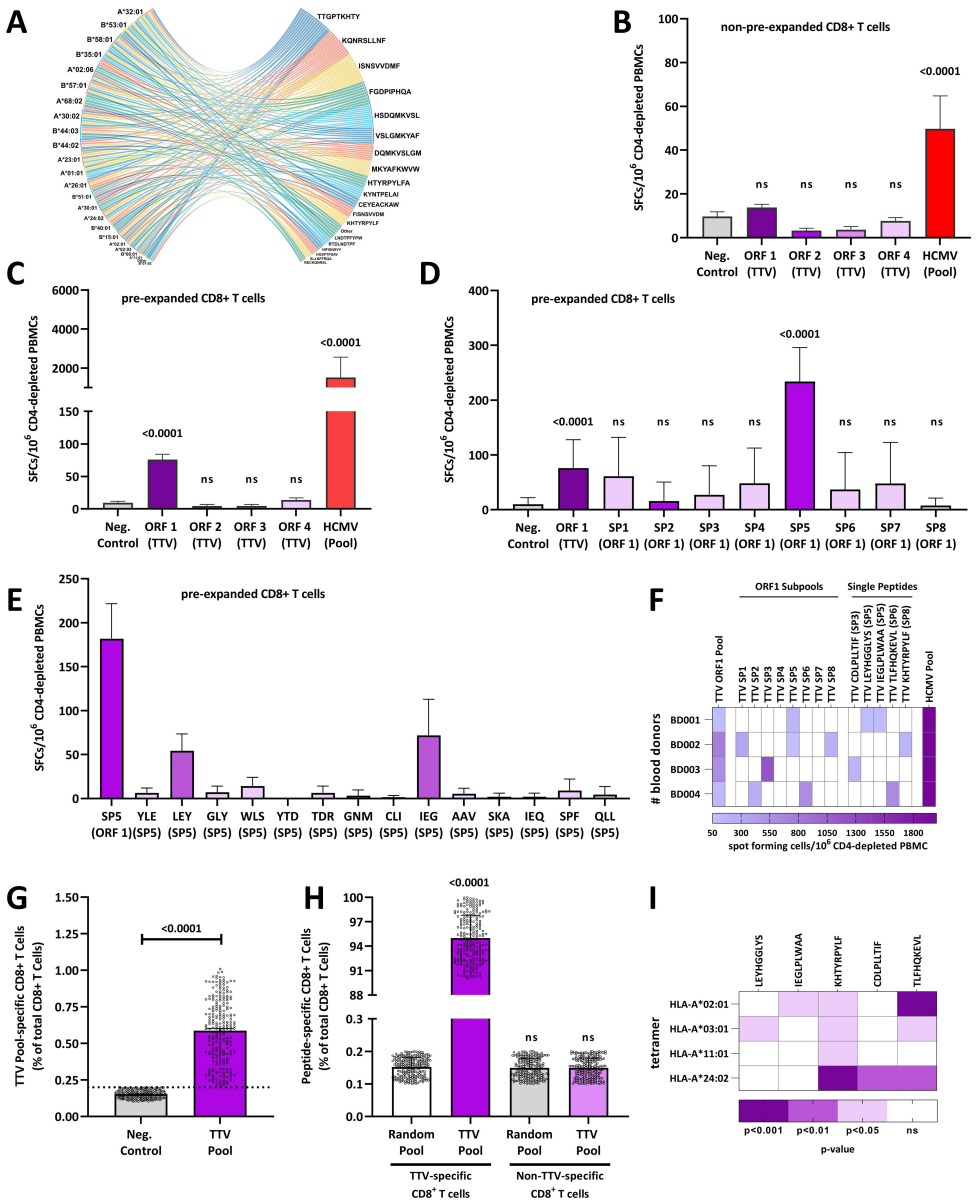
Torque teno viruses elicit TTV-specific CD8^+^ T-cell responses: **(A)** the cord diagram shows the top 0.02% peptides of ORF1–4 predicted to bind to the 27 HLA alleles of the reference set. **(B)** Individual CD8^+^ T-cell responses are shown for one blood donor (BB001) to TTV ORF1–4 or HCMV peptide pools measured in *ex vivo* IFNγ ELISPOT assays with non-pre-expanded CD8^+^ T cells. **(C–E)** Individual CD8^+^ T-cell responses of one blood donor (BB001) to **(C)** TTV ORF1–4 or HCMV peptide pools, **(D)** TTV sub-pools (SP1–8) and **(E)** single TTV peptides measured with TTV or HCMV peptide pools pre-expanded CD8^+^ T cells and IFNγ ELISPOTs, respectively. **(F)** Heat map of CD8^+^ T-cell responses to HCMV or TTV ORF1 pools, sub-pools, and single peptides in four healthy blood donors (BD001–BD004) measured with pre-expanded CD8^+^ T cells and IFNγ ELISPOT. White: No TTV-specific CD8^+^ T-cell response detected. **(G)** Frequencies of TTV-specific CD8^+^ T cells in 240 healthy blood donors (BD005–249) were assessed by a tetramer pool that consisted of HLA-A*02:01, HLA-A*03:01, HLA-A*11:01, and HLA-A*24:02 tetramers, loaded with a peptide pool that consists of the TTV-derived LEYHGGLYS, IEGLPLWAA, KHTYRPYLF, CDLPLLTIF, and TLFHQKEVL peptides. Data are shown as the percentage of TTV-specific CD8^+^ T cells of total CD8^+^ T cells. The dashed line represents the maximum response of the non-peptide control. Each dot represents one individual blood donor. **(H, I)** Fine specificity of the TTV-specific CD8^+^ T cell response: **(H)** TTV-specific CD8^+^ T cells or non-TTV-specific CD8^+^ T cells from 240 healthy blood donors (BD005–249) were first stained with TTV peptide-loaded APC-conjugated HLA tetramers and then sorted and re-stained with a PE-tetramer pool that consists of HLA-A*02:01, HLA-A*03:01, HLA-A*11:01, and HLA-A*24:02 tetramers, loaded with either a peptide pool that consists of the TTV-derived LEYHGGLYS, IEGLPLWAA, KHTYRPYLF, CDLPLLTIF, and TLFHQKEVL peptides or a random nine amino acid peptide library. Data are shown as the percentage of tetramer-positive cells. **(I)** Total CD8^+^ T cells from 240 healthy blood donors (BD005–249) were pooled and stimulated with individual HLA-A*02:01, HLA-A*03:01, HLA-A*11:01, and HLA-A*24:02 tetramers, loaded with individual TTV-derived LEYHGGLYS, IEGLPLWAA, KHTYRPYLF, CDLPLLTIF, and TLFHQKEVL peptides. Heat map shows the p-value in comparison to the non-peptide control of 10 independent experiments, as assessed with the Kruskal–Wallis Test. Differences were assessed with the Kruskal–Wallis test in comparison to the **(B–D, G, I)** non-peptide control (Neg.Control) or **(H)** the random peptide control. HCMV, human cytomegalovirus; ORF, open-reading frame; PBMC, peripheral blood mononuclear cell; SP, sub-pool; TTV, Torque teno virus; ns, not significant.

For the characterization of TTV-specific CD8^+^ T-cell responses, we first recruited one healthy HCMV-IgG seropositive blood donor (BD001), in whom TTV-DNA was detected in plasma. In BD001, an infection with the TTV-BNI-700611 strain (GenBank: MH017571.1) of the *Alphatorquevirus* homin1 species was identified by Sanger sequencing. We then genotyped the donor’s individual classical HLA class I alleles and identified the HLA-A*02:01, HLA-A*32:01, HLA-B*13:02, HLA-B*40:303, HLA-C*02:02, and HLA-C*06:02. Based on these data, *in silico* epitope predictions with the ORF1–4 of the assigned TTV strain and the donor’s individual HLA class I alleles were performed and yielded 124 peptides (**Supplementary Table S1**). Most peptides were located in ORF1 (N=89), while the remaining peptides were distributed between ORF2 and ORF4 (**Supplementary Table S1**). We also predicted the binding affinity across a reference set of HLA-I alleles, covering 97% of the human population and found that 77% of the same 124 TTV peptides, predicted for donor BD001, bind on average to six alleles of the HLA reference set and to up to 18 alleles per peptide. All HLA-I alleles included were predicted to bind at least one peptide but bound on average 16 peptides of the donor TTV strain per allele (**Figure 1A**).

To characterize the TTV-specific CD8^+^ T-cell responses, we stimulated CD4-depleted PBMCs from donor BD001 with individual ORF1–4 peptide pools and tested the stimulated cells in IFN-γ ELISpot assays. To compare the level of the responses with those of HCMV, we performed the assay also with an HCMV peptide pool that contains 28 HLA-I-restricted peptides. While BD001 showed robust activation of HCMV-specific CD8^+^ T cells, none of the TTV ORF peptide pools elicited detectable CD8^+^ T cell responses (**Figure 1B**).

Based on these results, we hypothesized that functional TTV-specific responses are scarce in contrast to HCMV-specific CD8^+^ T-cell responses. Therefore, we expanded CD4-depleted PBMCs with TTV ORFs peptide pools and tested the expanded cells again in IFN-γ ELISpot assays. As shown in **Figure 1C**, the TTV ORF1 peptide pool elicited detectable CD8^+^ T-cell responses in pre-expanded cells, whereas the peptides corresponding to TTV ORF2–4 pools induced no detectable responses. We further investigated in detail the peptides in the TTV ORF1 pool and conducted ELISpot assays using peptide sub-pools (SP1–8, **Supplementary Table S1**). One sub-pool (SP5) gave positive results (**Figure 1D**) and further tests with the constituent single peptides followed. Thereby, we identified two individual SP5 peptides (LEYHGGLYS and IEGLPLWAA) that induced detectable CD8^+^ T cell responses (**Figure 1E**).

To confirm these data, we analyzed three additional TTV-DNA and HCMV-seropositive blood donors (BD002-BD004) for their TTV-specific CD8^+^ T-cell responses. The strains of these donors were characterized by Sanger sequencing as the TTV P19-3 (GenBank: KT163917.1), tth31 (GenBank: AJ620225.1), and P10-c7 (GenBank: MW455443), all belonging to *Alphatorquevirus* homin3 species. As shown in **Figure 1F**, all donors showed a response against the ORF1 peptide pool, while different peptides (KHTYRPYLF, CDLPLLTIF, or TLFHQKEVL) were identified for each donor.

### TTV-specific CD8^+^ T-cell responses are universally detected in TTV-infected individuals

We next analyzed whether the five identified TTV-encoded peptides (LEYHGGLYS, IEGLPLWAA, KHTYRPYLF, CDLPLLTIF, and TLFHQKEVL) elicit a universally detectable TTV-specific immune response in TTV PCR-positive blood donors. Therefore, we recruited a study cohort of additional 120 healthy TTV-DNA-positive and HCMV-seropositive healthy blood donors (BD005–BD124) and 120 healthy TTV-DNA-positive and HCMV-seronegative healthy blood donors (BD125–BD249). We then isolated CD8^+^ T cells from the 240 blood donors and incubated the cells with a HLA-A tetramer pool (HLA-A*02:01, HLA-A*03:01, HLA-A*11:01, and HLA-A*24:02), loaded with a peptide pool that consists of the five previously identified TTV-derived peptides LEYHGGLYS, IEGLPLWAA, KHTYRPYLF, CDLPLLTIF, and TLFHQKEVL. We then determined the frequency of TTV peptide-pool-specific CD8^+^ T cells by flow cytometry. As shown in **Figure 1G**, TTV peptide-pool-specific CD8^+^ T cells were readily detectable in all blood donors (median, 0.6% of total CD8^+^ T cells), although some donors only showed low TTV peptide-pool-specific CD8^+^ T-cell frequencies.

To corroborate these findings, total CD8^+^ T cells from all 240 HCMV-seropositive and HCMV-seronegative donors were first stained with TTV peptide-loaded APC-conjugated HLA tetramers and then sorted into TTV-specific and non-specific CD8^+^ T cells. The sorted cells were then re-stained with PE-tetramers presenting either TTV peptides or random nine amino acid peptides. As shown in **Figure 1H**, only TTV-specific CD8^+^ T cells featured specific binding of the TTV tetramers, while, in contrast, no binding of TTV tetramers to the non-specific CD8^+^ T cells was observed. To complement tetramer staining with TTV peptide pools, we also carried out assays with individual HLA-A*02:01, HLA-A*03:01, HLA-A*11:01, and HLA-A*24:02 tetramers, loaded with single LEYHGGLYS, IEGLPLWAA, KHTYRPYLF, CDLPLLTIF, and TLFHQKEVL TTV peptides and pooled CD8^+^ T cells from all donors. As shown in **Figure 1I**; **Supplementary Figure S1**, the TLFHQKEVL and KHTYRPYLF peptides led to a response to three or four tested HLA tetramers, respectively, while the LEYHGGLYS, IEGLPLWAA, and CDLPLLTIF peptides bound to only one tested HLA-A tetramer.

Thus, TTV-infected individuals mount a universally detectable, but highly specific TTV-specific CD8^+^ T-cell response.

### TTV-specific CD8^+^ T cells mostly show an exhausted, non-functional phenotype

**Figure 2:**
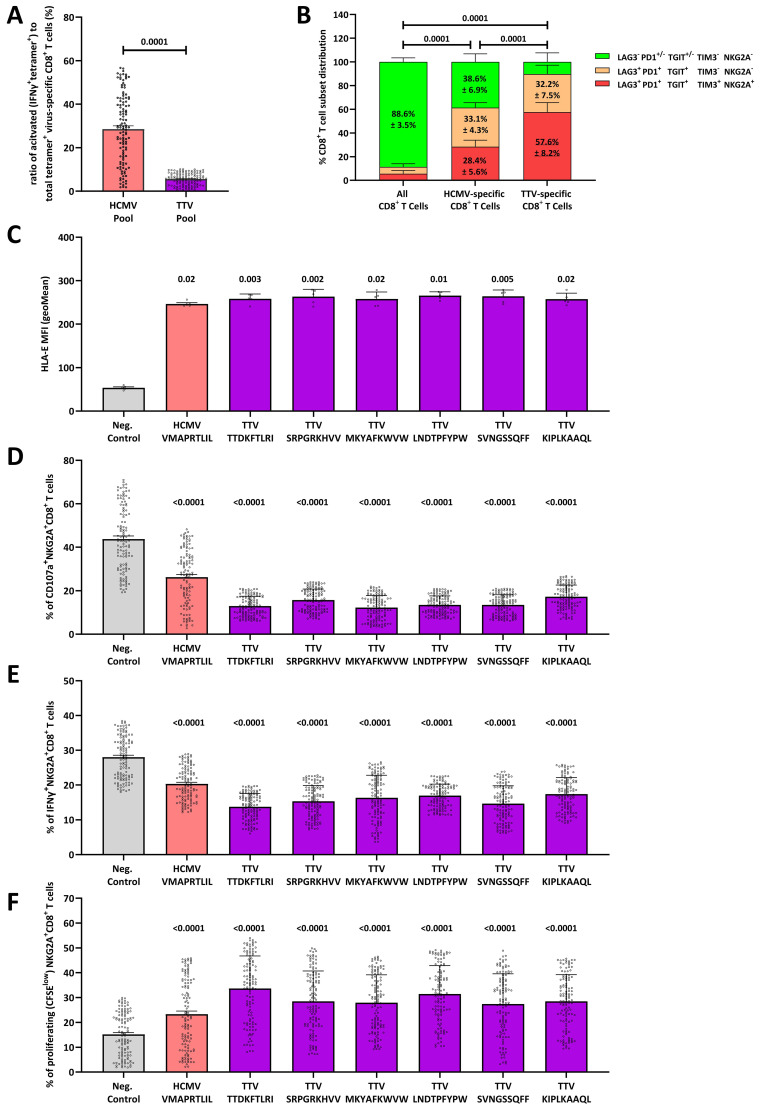
The HLA-E-TTV-NKG2A axis exhaust TTV-specific immune responses: **(A)** box-plot shows the percentage of activated (IFNγ^+^Tetramer-APC^+^) cells to virus-specific (Tetramer-APC^+^) CD8^+^ T cells in N=120 healthy TTV-DNA-positive and HCMV-seropositive healthy blood donors (BD005-BD124) and 120 healthy TTV-DNA-positive and HCMV-seronegative healthy blood donors (BD125–BD249) in response to an HCMV- or TTV-derived peptide pool, respectively. TTV-specific CD8^+^ T-cell responses were assessed in N=120 healthy TTV-DNA-positive and HCMV-seropositive healthy blood donors (BD005–BD124) and 120 healthy TTV-DNA-positive and HCMV-seronegative healthy blood donors (BD125–BD249), while the HCMV-specific CD8^+^ T-cell responses were assessed in N=120 healthy TTV-DNA-positive and HCMV-seropositive healthy blood donors (BD005–BD124). The TTV peptide pool consists of the TTV-derived LEYHGGLYS, IEGLPLWAA, KHTYRPYLF, CDLPLLTIF, and TLFHQKEVL peptides. Each dot represents one individual blood donor. Groups were compared using the Mann–Whitney test. **(B)** CD8^+^ T-cell phenotype of total, HCMV-specific or TTV-specific CD8^+^ T cells, respectively. The phenotype of total and TTV-specific CD8^+^ T cells was assessed in N=120 healthy TTV-DNA-positive and HCMV-seropositive healthy blood donors (BD005–BD124) and 120 healthy TTV-DNA-positive and HCMV-seronegative healthy blood donors (BD125–BD249). The phenotype of HCMV-specific CD8^+^ T cells was assessed in N=120 healthy TTV-DNA-positive and HCMV-seropositive healthy blood donors (BD005–BD124). Each fraction represents the mean frequency of the LAG3^−^ PD1^+/−^ TGIT^+/−^ TIM3^−^ NKG2A^−^, LAG3^+^ PD1^+^ TGIT^+^ TIM3^−^ NKG2A^−^ and LAG3^+^ PD1^+^ TGIT^+^ TIM3^+^ NKG2A^+^ CD8^+^ T-cell subsets (± SD). Groups were compared using the two-way ANOVA. **(C)** HLA-E stabilization assay: RMA/S-HLA-E cells were incubated together with 300 µM of the HCMV-encoded VMAPRTLIL or the TTV-encoded TTDKFTLRI, SRPGRKHVV, MKYAFKWVW, LNDTPFYPW, SVNGSSQFF, or KIPLKAAQL peptides. The geometric mean of the HLE-E MFI was then assessed by flow cytometry. Box plot represents the mean (± SD) of five independent replicates. **(D–F)** NKG2A^+^ inhibition and proliferation assay: **(D, E)** K562-HLA-E*0103/0103 or **(F)** RMA/S-HLA-E cells were first incubated together with 300 µM of the HCMV-encoded VMAPRTLIL or the TTV-encoded TTDKFTLRI, SRPGRKHVV, MKYAFKWVW, LNDTPFYPW, SVNGSSQFF, or KIPLKAAQL peptides and then incubated together with pre-activated and enriched CD8^+^ T cells. The percentage of **(D)** CD107a^+^, **(E)** IFNγ^+^, or **(F)** proliferating (CFSE^low^) NKG2A^+^CD8^+^ T cells was assessed by flow cytometry. Plots represent the mean (± SD) of 240 independent biological replicates, reflecting N=120 healthy TTV-DNA-positive and HCMV-seropositive healthy blood donors (BD005–BD124) and 120 healthy TTV-DNA-positive and HCMV-seronegative healthy blood donors (BD125–BD249). Each dot represents one individual blood donor. Each peptide was compared to K562-HLA-E*0103/0103 cells without peptides using the Kruskal–Wallis test. HCMV, human cytomegalovirus; IFNγ, interferon γ; MFI, mean fluorescence intensity; TTV, Torque teno virus.

Despite the existing CD8^+^ T-cell response, TTV-DNA is frequently detected in infected healthy individuals. This is in contrast to latent HCMV infections, where virus reactivations are rarely observed and mainly controlled by human CD8^+^ T cell responses (28). Therefore, we next compared the TTV- and HCMV-specific CD8^+^ T-cell responses. We measured the TTV- and HCMV-specific CD8^+^ T-cell frequencies individually for each donor using TTV- and HCMV-peptide pool loaded tetramers and the cellular activation, as demonstrated by APC-Tetramer^+^IFNγ^+^ cells, in response to the TTV- and the HCMV-peptide pool loaded tetramers, respectively, by flow cytometry (**Supplementary Figure S2A**). As shown in **Figure 2A**, a median of 32.7% of the total HCMV-specific CD8^+^ T cells, derived from HCMV-seropositive blood donors, was also IFNγ positive and was thus activated in response to the HCMV peptides. In contrast, only 5.7% of the TTV-specific CD8^+^ T cells were also IFNγ positive in HCMV-seropositive and HCMV-seronegative blood donors, respectively. HCMV-seropositive and HCMV-seronegative healthy blood donors showed, however, no significant differences in the frequency of TTV peptide-pool-specific CD8^+^ T cell levels.

We then analyzed HCMV- and TTV-specific CD8^+^ T cells for the expression of LAG3, PD1, and TGIT, which reflect a repeated cellular stimulation, and the expression of TIM3 and NKG2A, which represent a terminally exhausted and non-functional CD8^+^ T-cell phenotype (29). While the majority of HCMV-specific CD8^+^ T cells in HCMV-seropositive blood donors showed a LAG3^−^ PD1^+/−^ TGIT^+/−^ TIM^−^ NKG2A^−^ phenotype (**Figure 2B**), the non-functional and terminally exhausted LAG3^+^ PD1^+^ TGIT^+^ TIM^+^ NKG2A^+^ phenotype significantly dominated in TTV-specific CD8^+^ T cells.

### TTV-encoded peptides elicit potent NKG2A responses

As the majority of TTV-specific CD8^+^ T cells express NKG2A, we next hypothesized that a so far unknown TTV-encoded peptide binds and stabilizes HLA-E on the surface of TTV-infected cells, thereby leading to stimulation and upregulation of NKG2A on the surface of TTV-specific CD8^+^ T cells. To test our hypothesis, we predicted HLA-E-binding peptides in the TTV-BNI-700611 strain, found in the initial blood donor BD001 (**Supplementary Table S1**). We then tested all predicted peptides (N=230) in an HLA-E stabilization assay that assesses the peptide-dependent upregulation of HLA-E on RMA-S/HLA-E/LFA-3 cells. Six peptides (TTDKFTLRI, SRPGRKHVV, MKYAFKWVW, LNDTPFYPW, SVNGSSQFF, and KIPLKAAQL) led to a significant HLA-E upregulation; the remaining TTV-encoded peptides had no effect on the HLA-E stabilization (**Figure 2C**; **Supplementary Figure S3**).

To further assess the capacity of these peptides to inhibit all NKG2A^+^ cells, we next tested these peptides in NKG2A^+^CD8^+^ T cell, NKG2A^+^CD4^+^ T cell, and NKG2A^+^CD56^+^ NK cell inhibition and proliferation assays (**Supplementary Figure S2B**) in cells, derived from all N=240 blood donors. All six peptides led to significant inhibition of the cellular cytotoxicity, as determined by fewer CD107a^+^NKG2A^+^CD8^+^ T cells (**Figure 2D**) and CD107a^+^NKG2A^+^NK cells (**Supplementary Figure S4**). All led to a reduced IFNγ secretion in NKG2A^+^CD8^+^ T cells (**Figure 2E**), NKG2A^+^ NK cells (**Supplementary Figure S4**), and NKG2A^+^CD4^+^ T cells (**Supplementary Figure S4**) and a strong proliferation of NKG2A^+^CD8^+^ T cells (**Figure 2F**), NKG2A^+^ NK cells (**Supplementary Figure S4**) and NKG2A^+^ CD4^+^ T cells (**Supplementary Figure S4**).

### TTV-peptides are highly polymorphic

**Figure 3:**
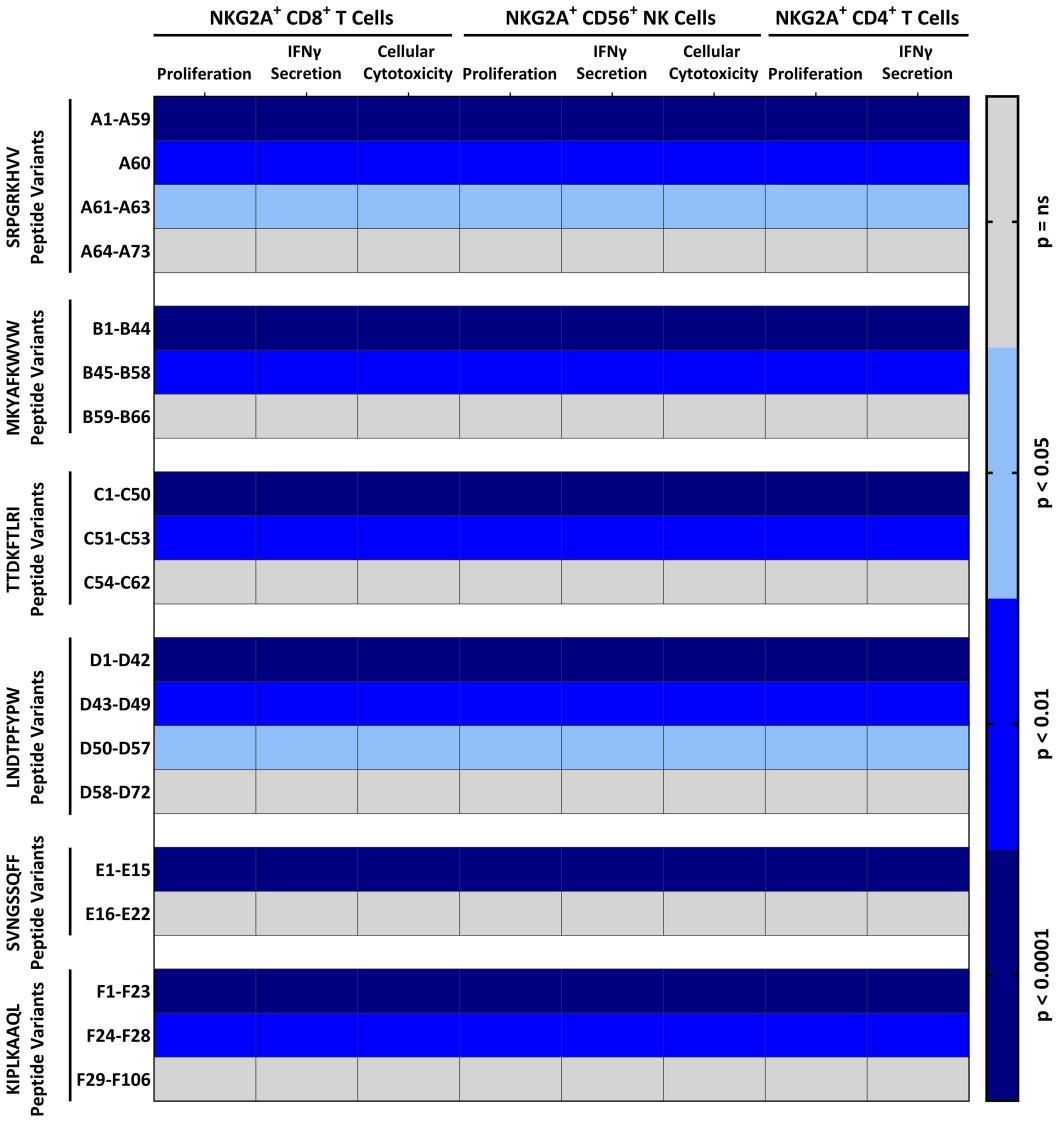
Distinct TTV-peptide variants elicit potent NKG2A^+^-mediated effector functions: NKG2A^+^ inhibition and proliferation assay: K562-HLA-E*0103/0103 or RMA/S-HLA-E cells were first incubated together with 300 µM of indicated TTV-encoded peptide variants (**Supplementary Table S2**) and then incubated together with pre-activated and enriched CD8^+^ T cells, CD56^+^ NK cells, or CD4^+^ T cells. The percentage of cytotoxic CD107a^+^NKG2A^+^CD8^+^ T cells or CD107a^+^NKG2A^+^CD56^+^ NK cells, and IFNγ^+^NKG2A^+^CD8^+^ T cells, IFNγ^+^NKG2A^+^CD4^+^ T cells, or IFNγ^+^NKG2A^+^CD56^+^ NK cells, and proliferating (CFSE^low^) NKG2A^+^CD8^+^ T cells, NKG2A^+^CD4^+^ T cells, or NKG2A^+^CD56^+^ NK cells was assessed by flow cytometry. Heat map shows the p-value in comparison to the non-peptide control of 240 independent biological replicates, reflecting N=120 healthy TTV-DNA-positive and HCMV-seropositive healthy blood donors (BD005–BD124) and 120 healthy TTV-DNA-positive and HCMV-seronegative healthy blood donors (BD125–BD249). Peptides were numbered after NKG2A^+^ inhibition and proliferation assays according to the statistical difference in comparison to the non-peptide control. IFNγ, interferon γ.

As TTV is a highly polymorphic virus, we next analyzed whether the six HLA-E stabilizing TTV peptides exist in different variants in various TTV strains and whether variations in the number of stabilizing peptides influence the HLA-E-mediated inhibition and proliferation of NKG2A^+^ cells. Therefore, we analyzed 199 already published sequenced TTV strains, which cover all 20 TTV species for the SRPGRKHVV, MKYAFKWVW, TTDKFTLRI, LNDTPFYPW, SVNGSSQFF, and KIPLKAAQL peptide variants (**Supplementary Table S2**). We identified in total 402 variants of the six peptides (SRPGRKHVV variants, A1–A73; MKYAFKWVW variants, B1–B66; TTDKFTLRI variants, C1–C62; LNDTPFYPW variants, D1–D72; SVNGSSQFF variants, E1–E22; and KIPLKAAQL variants, F1–F106), with most variants in the KIPLKAAQL peptide. We then tested all 402 variants in NKG2A^+^CD8^+^ T cell, NKG2A^+^CD4^+^ T cell, and NKG2A^+^CD56^+^ NK cell inhibition and proliferation assays in cells, derived from all 240 blood donors (BD005–249). As shown in **Figure 3**; **Supplementary Figure S5**, a total of 274 (68.2%) peptide variants led to the inhibition and proliferation of NKG2A^+^CD8^+^ T cells, NKG2A^+^CD4^+^ T cells, and NKG2A^+^CD56^+^ NK cells.

We then also analyzed the presence of these functional-inhibitory TTV peptide variants in the 199 published TTV sequences. The TTV strains encoded for mean 3.7 inhibitory TTV peptide variants. Most peptides were encoded by TTV *Alphatorquevirus* homin1 and 21 (mean ± SD: 4.3 ± 1.2 and 4.1 ± 0.4 inhibitory TTV peptide variants/strain, respectively), while TTV *Alphatorquevirus* homin29 and 6, exhibited the least peptides (mean ± SD: 2.7 ± 1.1 and 2.3 ± 1.7 inhibitory TTV peptide variants/strain, respectively).

### Inhibitory TTV peptides variants are associated with an increased TTV replication

**Figure 4:**
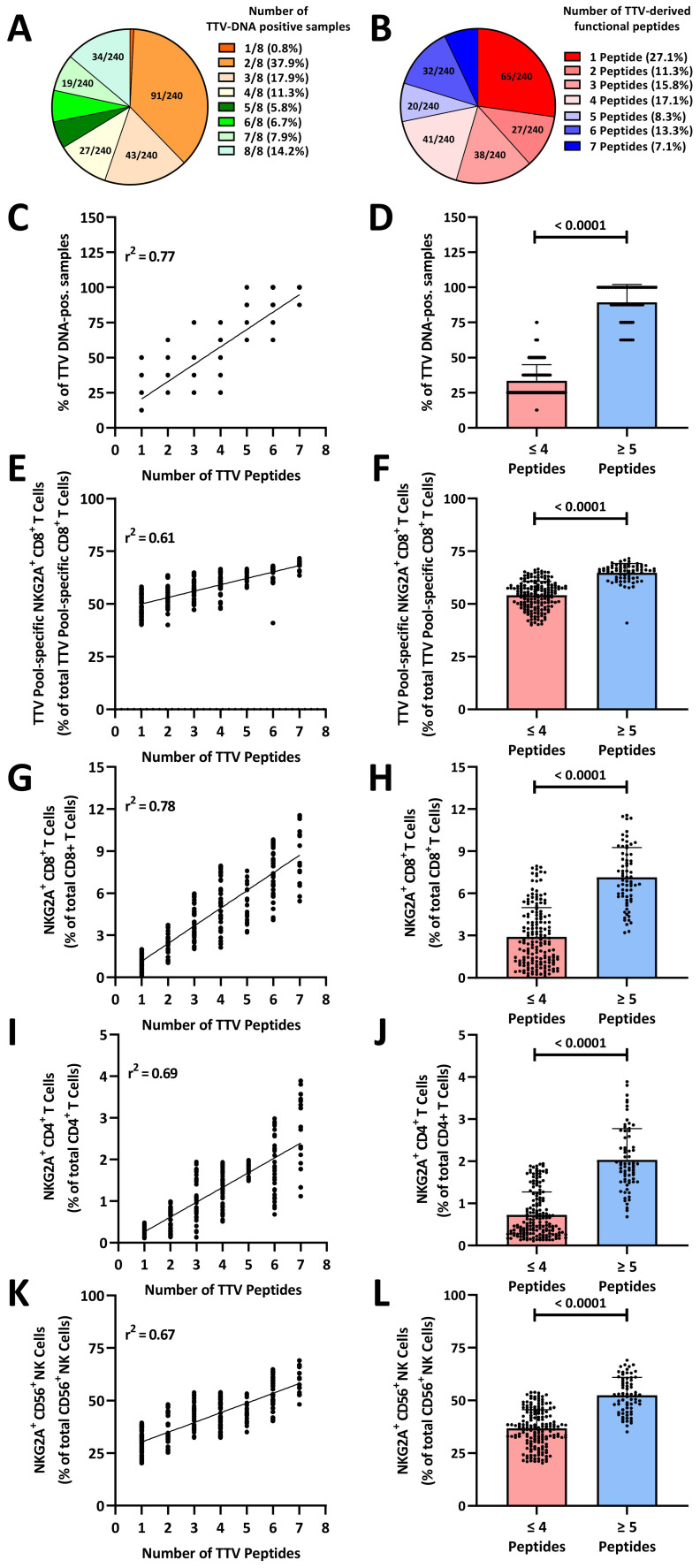
TTV-peptide variants imprint the human immune system: **(A)** number of TTV-DNA positive samples in healthy blood donors. Seven follow-up plasma samples from N=240 healthy TTV-DNA-positive healthy blood donors (BD005–BD249) were collected in 6-month (± 3.5 week) intervals. Fractions represent the relative frequency of TTV-DNA-positive samples. **(B)** Number of functional TTV peptides. TTV sequences of N=240 healthy TTV-DNA-positive healthy blood donors (BD005–BD249) were analyzed by next-generation sequencing and blasted against known TTV peptide variants (**Supplementary Table S2**). Fractions represent the relative frequency of the number of functional TTV-derived peptides (**Figure 3**). **(C–L)** The number of functional TTV-derived peptides in N=240 healthy blood donors was compared to **(C, D)** the frequency of TTV-DNA positive samples, **(E, F)** TTV-pool (LEYHGGLYS, IEGLPLWAA, KHTYRPYLF, CDLPLLTIF, and TLFHQKEVL)-specific NKG2A^+^CD8^+^ T cells, **(G, H)** total NKG2A^+^CD8^+^ T cells, **(I, J)** total NKG2A^+^CD4^+^ T cells, **(K, L)** total NKG2A^+^CD56^+^ NK cells. Associations were assessed by **(C, E, G, I, K)** linear regression. **(D, F, H, J, L)** Box plots: each dot represents one individual blood donor. Box plots represent the mean (± SD) of 240 independent biological replicates. Groups were compared using the Mann–Whitney test. TTV, Torque teno virus.

To analyze the impact of these TTV peptide variants on the extent of the viral replication, we collected seven follow-up plasma samples in a 6-month interval from all 240 blood donors (BD005–249) and tested all samples from each individual for TTV-DNA. As shown in **Figure 4A**, the data widely differed between the donors. In 0.8% of the blood donors, only the first sample was tested positive for TTV-DNA, while in 14.2%, TTV-DNA was identified in all follow-up samples obtained from a single blood donor. Of all blood donors with more than one TTV-positive sample, 76.8% (N=183) were intermittent TTV DNA-positive, as defined by TTV DNA-negative between TTV DNA-positive time points. In contrast, only 23.1% (N=55) of all blood donors with more than one TTV-positive sample were continuously TTV DNA positive. The frequency of TTV DNA-positive samples was, however, not significantly different between the individual time points.

We then sequenced the TTV strains identified in the first plasma sample by next-generation sequencing and analyzed all TTV reads for their HLA-E-stabilizing peptides by BLAST. As shown in **Figure 4B**, between one and seven inhibitory, HLA-E-stabilizing TTV peptide variants were identified in the blood donors. In 69 (28.8%) blood donors, five or more inhibitory TTV peptides were identified. We then correlated the percentage of TTV-DNA-positive plasma samples with the number of inhibitory TTV peptides detected in the first viremic sample of each donor. As shown in **Figure 4C**, the number of HLA-E-stabilizing TTV peptides in the first sample showed a significant correlation with the number of TTV-DNA-positive plasma samples in the follow-up. The presence of ≥5 TTV-derived inhibitory peptides was significantly associated with a substantially increased frequency of TTV-DNA-positive samples (**Figure 4D**). To analyze, whether the number of peptides is associated with the exhaustion of TTV-specific CD8^+^ T cells, we correlated the frequency of NKG2A^+^ TTV-specific CD8^+^ T cells with the number of inhibitory TTV peptides. As shown in **Figure 4E**, we found a significant positive correlation between the frequency of NKG2A^+^ TTV-specific CD8^+^ T cells and the number of inhibitory TTV peptides. The presence of ≥5 inhibitory TTV-derived peptides was significantly associated with higher frequencies of terminally exhausted NKG2A^+^ TTV-specific CD8^+^ T cells (**Figure 4F**).

### TTV leads to a stable imprint in the human immune system

Finally, we analyzed to which extent these TTV-encoded peptides shape the overall NKG2A^+^ human immune responses. We therefore correlated the number of inhibitory TTV-derived peptides with the frequency of total NKG2A^+^ cells. We found a significant correlation between the number of inhibitory TTV peptides and the frequency of total NKG2A^+^CD8^+^ T cells (**Figure 4G**), NKG2A^+^CD4^+^ T cells (**Figure 4I**), and NKG2A^+^CD56^+^ NK cells (**Figure 4K**). The presence of ≥5 inhibitory TTV peptides was significantly associated with high levels of NKG2A^+^CD8^+^ T cells (**Figure 4H**), NKG2A^+^CD4^+^ T cells (**Figure 4J**), and NKG2A^+^CD56^+^ NK cells (**Figure 4L**).

## Discussion

Here, we report that TTV elicits a functional, but highly exhausted TTV-specific CD8^+^ T-cell response, which is hallmarked by the inhibitory NKG2A-receptor expression and that TTV imprints the human immune system towards NKG2A^+^ cells.

Our study revealed that TTV elicits a universally detectable TTV-specific CD8^+^ T-cell response. This is in line with previous studies, which suggested that the TTV persistence is limited due to constant immune surveillance (30). These data raise the question of how TTV can establish chronically persistent infections and generate a minimum of approximately 3.8 × 10^10^ virions per day in each individual, despite the generation of a TTV-specific CD8^+^ T-cell response (30). Here, we demonstrate that the vast majority of overall TTV-specific CD8^+^ T cells are, in fact, non-functional and consist of an exhausted LAG3^+^ PD1^+^ TGIT^+^ TIM3^+^ NKG2A^+^ phenotype. This exhausted phenotype was recently described as a result of repeated stimulations and cell divisions of CD8^+^ T cells (29). When comparing the CD8^+^ T-cell populations directed against TTV and HCMV, which is only sporadically replicating to a higher level, we found that there were significantly fewer non-functional and LAG3^+^ PD1^+^ TGIT^+^ TIM3^+^ NKG2A^+^ cells in the subset of HCMV-specific CD8^+^ T cells. This may indicate that the TTV replication is, in contrast to HCMV, less efficiently controlled by virus-specific CD8^+^ T cells and, as a result, TTV-DNA is frequently detectable in the blood of healthy individuals (28).

In the search for the functional basis of our findings, we identified six peptides that upregulate and stabilize HLA-E on the cell surface, stimulate the NKG2A receptor, and inhibit the activation of NKG2A^+^CD8^+^ T and NKG2A^+^ NK cells. As a result, TTV may evade the human cellular antiviral cytotoxic immune responses. In our study cohort, the number of inhibitory HLA-E stabilizing peptides present in a single host, provided either by one or by the mixture of different TTV strains, was significantly correlated with the presence of TTV viremia and exhausted, non-functional TTV-specific CD8^+^ T cells. This suggests that the inhibition of NKG2A^+^ TTV-specific CD8^+^ T cells by inhibitory TTV-derived peptides may contribute to persistent viremia. This is in line with previously published studies that demonstrated that a potent inhibition of NKG2A^+^ cells via high-affinity HCMV-encoded UL40 peptides is a risk factor for the development of recurring high-level HCMV episodes in transplant recipients (31). As our study was, however, limited by the absence of established TTV *in vitro* or *in vivo* models (4), further studies are needed to analyze the impact of distinct TTV-encoded inhibitory peptide variants on the exhaustion of NKG2A^+^ TTV-specific CD8^+^ T cells and the TTV replication in the human host.

The HLA-E/NKG2A axis was recently identified as one important immune evasive mechanism in other viral infections such as HCMV, Epstein–Barr virus (EBV), human immunodeficiency viruses (HIV), herpes simplex virus 1 (HSV-1), or human papillomavirus (HPV) (32, 33). With all these viral infections, a multitude of other immune evasive mechanisms have been shown to impair distinct components of the virus-specific immune response and contribute to the establishment of life-long chronically persistent or latent infections (34–37). Additional studies are therefore needed to explore potential additional mechanisms of TTV-mediated immune evasion.

As another major finding, we revealed that, on a functional level, distinct TTV-encoded peptide variants lead to the proliferation of NKG2A^+^ cells. We demonstrate that a higher number of functional TTV-derived peptides was significantly associated with a higher frequency of NKG2A^+^CD8^+^ T cells, NKG2A^+^CD4^+^ T cells, and NKG2A^+^ NK cells.

These data are of special interest, as some studies identified a high frequency of NKG2A^+^ cells, and the high-level NKG2A expression, as a risk factor for the development and the prognosis of distinct malignant diseases, and for the severity of distinct viral infections, such as chronic hepatitis B, or with severe coronavirus disease 2019 (COVID-19) (32). On the contrary, some studies recently associated low NKG2A^+^ cell frequencies or low-level NKG2A expression with an increased risk and a worse clinical prognosis of autoimmune diseases, such as systemic lupus erythematosus, psoriasis, and rheumatoid arthritis (32, 38). Based on these results, it has been speculated that NKG2A^+^ cells have an important function in balancing the human immune system to avoid a hyper- or hyporeactive immune response (32).

Importantly, our present study thus suggests that TTV may have a substantial imprint on the human immune system that, based on the TTV- peptide driven NKG2A^+^-mediated regulation, may have a substantial impact on the development of other diseases. Further analyses are needed to clarify whether a distinct TTV population in an individual host has an impact on the development or course of different infectious, autoimmune, and malignant diseases.

As a limitation of our study, all HLA peptide predictions were based on one reference TTV *Alphatorquevirus* homin1 species that was infecting one reference blood donor. Other TTV species or TTV strains infecting individuals with different HLA alleles may elicit additional, so far undefined, TTV-specific T-cell responses. Future studies are thus required to further elucidate the TTV-specific immune responses in individual hosts.

In summary, we show that TTV infections result in a functional but highly exhausted TTV-specific CD8^+^ T-cell response and that there may be a TTV-mediated imprint on the human immune system towards NKG2A^+^ cells. Further studies are required to confirm whether the characterization and determination of these functionally relevant peptides in the individual host may serve as a prognostic biomarker for the development and progression of a variety of different NKG2A-associated diseases.

## Data Availability

The raw data supporting the conclusions of this article will be made available by the authors, without undue reservation. The data presented in the study are deposited in the figshare repository, accession number DOI: 10.6084/m9.figshare.26807947.
