# Supplementary material for: Torque teno viruses exhaust and imprint the human immune system via the HLA-E/NKG2A axis
Source: Front Immunol. 2024 Sep 4;15:1447980. doi: 10.3389/fimmu.2024.1447980 (PMC11408220; doi:10.3389/fimmu.2024.1447980)
Supplement: Supplementary file 1 [file DataSheet1.pdf]

# Supplementary Information

Table S1: TTV peptides predicted for HLA-I alleles and the TTV strain of blood donor BD001.......... 2

Table S2: Overview to TTV-derived Peptide Variants .......................................................................... 7

Figure S1 ......................................................................................................................... 27

Figure S2 ......................................................................................................................... 28

Figure S3 ......................................................................................................................... 30

Figure S4 ......................................................................................................................... 32

Figure S5 ......................................................................................................................... 34

Table S1: TTV peptides predicted for HLA-I alleles and the TTV strain of blood donor BD001

| **TTV-BNI-700611-G4-CSF**<br>Accession Number: MH017571 | | |
|---|---|---|
| **Open Reading Frame** | **Accession Number** | **Amino Acid Sequence** |
| ORF1 | A0A3S8RKH8 | MAYGWWARRRRRWRRWRRRPWRRRWRTRRRRPRRRYRRRR<br>HVRRRRRGRWRRRYRKWRRKGRRRGKKKIIIRQWQPNYRRRC<br>NIVGYMPVLICGNNTVCRNYATHSDDSYLPGPFGGGMTTDKFT<br>LRILYDEYKRFMNYWTASNEDLDLCRYRGCTLWFFRHPEVDFI<br>ILINTMPPFLDTQLTGPSIHPGLMALNKRARWIPSLKSRPGRKHV<br>VKIRVGAPRMFTDKWYPQSDLCDLPLLTIFASAADMQYPFGSP<br>LTDTVVVGFQVLQSMYNDCLSILPENFNNGGKGKALHEKITQY<br>LPNYNTTQTLAQLKPYIDNTSTTTTNNWSSYINTTKFTTASQPT<br>TNSGAAPYYTFADTWYRGTAYNTAITKIPLKAAQLYDNTTKKL<br>LGTTFTGGSPYLEYHGGLYSSIWLSAGRSYFETKGTYTDVTYNP<br>FTDRGEGNMVWIDWVSKSDSVYSKTQSKCLIEGLPLWAAVYG<br>YAEYCSKATGDTNIEQNCRVVIRSPFTNPQLLDHNNPLRGYVPY<br>SLNFGNGKMPGGSSQVPIRMRSKWYPTLFHQKEVLEAIAQGGP<br>FAYHSDQMKVSLGMKYAFKWVWGGNPVSQQVVRNPCKDTG<br>VSSGNRVPRSVQIVDPKYNTPELAIHAWDFRRACLAQKLLREC<br>KQNRSLLNFFRQAEKDTGETQRLCSPAKKSSKKKTYLSSQSSSS<br>DQAPLGGVGPKPKRGRGSPTRDADALPAAPAAAQGAAAHGSP<br>TPSAVPTITTGPTKHTYRPYLFAKGGGRNIFISNSVVDMFGDPIP<br>HQATSQDWQCEYEACKAWDRPARTDLNDTPFYPWAPRPKNP<br>YSVCFRLGFK |
| ORF2 | A0A3S8RKI2 | MHFSRIQRKKRLLLLQTLPASKKARQLLTGMWSPPTEDERVRE<br>RKWLLSVFQSHSAFRGCNDPVGHLCRLATLSNRPENPGPSGGP<br>RTPQIRRLPALPAAPQEPGDRAPWPMGGGPGDADAGAAGDAG<br>PGDADGGPADADLVAAIDAADM |
| ORF3 | A0A451ENP3 | MQTEPFSTELLSPGRKRYRRDTEALLPSQEEQQKENLSFLPVKQ<br>LRPSPPWRSRTKAKARKRVSNKRRRRSPSSSSSSSRSSSSWESNS<br>ERCSNNYNGSNKTHISTLPFCQGGRA |
| ORF4 | A0A3S8RKG4 | MNVSVSVNGSSQFFSLTLLSVAAMILSVTFVAWLPCLTVRRTPA<br>PPEDPVLLRSGAYPLSRLLPKSPVIEHHGLWVVGPETQTLAPLE<br>TQALETPMEDPQTQTSSPL |

| **Maxi Peptide Pool ORF 1** | | | |
|---|---|---|---|
| **Subpool** | **Index** | **AA Start** | **Amino Acid Sequence** |
| **ORF 1 SP 1** | 1 | 34 | RRYRRRRHV |
| **ORF 1 SP 1** | 2 | 72 | RQWQPNYRR |
| **ORF 1 SP 1** | 3 | 84 | IVGYMPVLI |
| **ORF 1 SP 1** | 4 | 90 | VLICGNNTV |
| **ORF 1 SP 1** | 5 | 120 | TTDKFTLRI |
| **ORF 1 SP 1** | 6 | 121 | TDKFTLRIL |
| **ORF 1 SP 1** | 7 | 128 | ILYDEYKRF |
| **ORF 1 SP 1** | 8 | 131 | DEYKRFMNY |
| **ORF 1 SP 1** | 9 | 136 | FMNYWTASN |
| **ORF 1 SP 2** | 10 | 156 | TLWFFRHPE |
| **ORF 1 SP 2** | 11 | 160 | FRHPEVDFI |
| **ORF 1 SP 2** | 12 | 161 | RHPEVDFII |
| **ORF 1 SP 2** | 13 | 163 | PEVDFIILI |
| **ORF 1 SP 2** | 14 | 165 | VDFIILINT |

| | | | |
|---|---|---|---|
| **ORF 1 SP 2** | 15 | 169 | ILINTMPPF |
| **ORF 1 SP 2** | 16 | 186 | SIHPGLMAL |
| **ORF 1 SP 2** | 17 | 190 | GLMALNKRA |
| **ORF 1 SP 2** | 18 | 192 | MALNKRARW |
| **ORF 1 SP 2** | 19 | 196 | KRARWIPSL |
| **ORF 1 SP 2** | 20 | 206 | SRPGRKHVV |
| **ORF 1 SP 3** | 21 | 232 | SDLCDLPLL |
| **ORF 1 SP 3** | 22 | 235 | CDLPLLTIF |
| **ORF 1 SP 3** | 23 | 243 | FASAADMQY |
| **ORF 1 SP 3** | 24 | 245 | SAADMQYPF |
| **ORF 1 SP 3** | 25 | 249 | MQYPFGSPL |
| **ORF 1 SP 3** | 26 | 253 | FGSPLTDTV |
| **ORF 1 SP 3** | 27 | 255 | SPLTDTVVV |
| **ORF 1 SP 3** | 28 | 257 | LTDTVVVGF |
| **ORF 1 SP 3** | 29 | 260 | TVVVGFQVL |
| **ORF 1 SP 3** | 30 | 263 | VGFQVLQSM |
| **ORF 1 SP 4** | 31 | 270 | SMYNDCLSI |
| **ORF 1 SP 4** | 32 | 291 | ALHEKITQY |
| **ORF 1 SP 4** | 33 | 297 | TQYLPNYNT |
| **ORF 1 SP 4** | 34 | 308 | TLAQLKPYI |
| **ORF 1 SP 4** | 35 | 323 | TTNNWSSYI |
| **ORF 1 SP 4** | 36 | 328 | SSYINTTKF |
| **ORF 1 SP 4** | 37 | 343 | TTNSGAAPY |
| **ORF 1 SP 4** | 38 | 346 | SGAAPYYTF |
| **ORF 1 SP 4** | 39 | 347 | GAAPYYTFA |
| **ORF 1 SP 4** | 40 | 360 | RGTAYNTAI |
| **ORF 1 SP 4** | 41 | 379 | YDNTTKKLL |
| **ORF 1 SP 4** | 42 | 385 | KLLGTTFTG |
| **ORF 1 SP 4** | 43 | 389 | TTFTGGSPY |
| **ORF 1 SP 5** | 44 | 397 | YLEYHGGLY |
| **ORF 1 SP 5** | 45 | 398 | LEYHGGLYS |
| **ORF 1 SP 5** | 46 | 403 | GLYSSIWLS |
| **ORF 1 SP 5** | 47 | 409 | WLSAGRSYF |
| **ORF 1 SP 5** | 48 | 423 | YTDVTYNPF |
| **ORF 1 SP 5** | 49 | 432 | TDRGEGNMV |
| **ORF 1 SP 5** | 50 | 437 | GNMVWIDWV |
| **ORF 1 SP 5** | 51 | 459 | CLIEGLPLW |
| **ORF 1 SP 5** | 52 | 461 | IEGLPLWAA |
| **ORF 1 SP 5** | 53 | 468 | AAVYGYAEY |
| **ORF 1 SP 5** | 54 | 478 | SKATGDTNI |
| **ORF 1 SP 5** | 55 | 486 | IEQNCRVVI |
| **ORF 1 SP 5** | 56 | 496 | SPFTNPQLL |
| **ORF 1 SP 5** | 57 | 502 | QLLDHNNPL |
| **ORF 1 SP 6** | 58 | 524 | KMPGGSSQV |
| **ORF 1 SP 6** | 59 | 528 | GSSQVPIRM |

| | | | |
|---|---|---|---|
| **ORF 1 SP 6** | 60 | 536 | MRSKWYPTL |
| **ORF 1 SP 6** | 61 | 537 | RSKWYPTLF |
| **ORF 1 SP 6** | 62 | 543 | TLFHQKEVL |
| **ORF 1 SP 6** | 63 | 546 | HQKEVLEAI |
| **ORF 1 SP 6** | 64 | 554 | IAQGGPFAY |
| **ORF 1 SP 6** | 65 | 563 | HSDQMKVSL |
| **ORF 1 SP 6** | 66 | 565 | DQMKVSLGM |
| **ORF 1 SP 6** | 67 | 569 | VSLGMKYAF |
| **ORF 1 SP 6** | 68 | 573 | MKYAFKWVW |
| **ORF 1 SP 6** | 69 | 582 | GGNPVSQQV |
| **ORF 1 SP 7** | 70 | 616 | KYNTPELAI |
| **ORF 1 SP 7** | 71 | 640 | RECKQNRSL |
| **ORF 1 SP 7** | 72 | 643 | KQNRSLLNF |
| **ORF 1 SP 7** | 73 | 647 | SLLNFFRQA |
| **ORF 1 SP 7** | 74 | 657 | KDTGETQRL |
| **ORF 1 SP 7** | 75 | 676 | KTYLSSQSS |
| **ORF 1 SP 7** | 76 | 686 | SDQAPLGGV |
| **ORF 1 SP 7** | 77 | 707 | RDADALPAA |
| **ORF 1 SP 7** | 78 | 725 | HGSPTPSAV |
| **ORF 1 SP 8** | 79 | 737 | TTGPTKHTY |
| **ORF 1 SP 8** | 80 | 742 | KHTYRPYLF |
| **ORF 1 SP 8** | 81 | 743 | HTYRPYLFA |
| **ORF 1 SP 8** | 82 | 756 | RNIFISNSV |
| **ORF 1 SP 8** | 83 | 757 | NIFISNSVV |
| **ORF 1 SP 8** | 84 | 759 | FISNSVVDM |
| **ORF 1 SP 8** | 85 | 760 | ISNSVVDMF |
| **ORF 1 SP 8** | 86 | 768 | FGDPIPHQA |
| **ORF 1 SP 8** | 87 | 783 | CEYEACKAW |
| **ORF 1 SP 8** | 88 | 796 | RTDLNDTPF |
| **ORF 1 SP 8** | 89 | 799 | LNDTPFYPW |
| **Maxi Peptide Pool ORF 2** | | | |
| **Subpool** | **Index** | **AA Start** | **Amino Acid Sequence** |
| **ORF 2** | 90 | 4 | SRIQRKKRL |
| **ORF 2** | 91 | 7 | QRKKRLLLL |
| **ORF 2** | 92 | 10 | KRLLLLQTL |
| **ORF 2** | 93 | 12 | LLLLQTLPA |
| **ORF 2** | 94 | 42 | RERKWLLSV |
| **ORF 2** | 95 | 49 | SVFQSHSAF |
| **ORF 2** | 96 | 86 | PRTPQIRRL |
| **ORF 2** | 97 | 137 | ADADLVAAI |
| **Maxi Peptide Pool ORF 3** | | | |
| **Subpool** | **Index** | **AA Start** | **Amino Acid Sequence** |
| **ORF 3** | 98 | 3 | TEPFSTELL |
| **ORF 3** | 99 | 18 | YRRDTEALL |

| | | | |
|---|---|---|---|
| **ORF 3** | 100 | 31 | EQQKENLSF |
| **ORF 3** | 101 | 32 | QQKENLSFL |
| **ORF 3** | 102 | 34 | KENLSFLPV |
| **ORF 3** | 103 | 37 | LSFLPVKQL |
| **ORF 3** | 104 | 43 | KQLRPSPPW |
| **ORF 3** | 105 | 77 | SSSRSSSSW |
| **ORF 3** | 106 | 99 | SNKTHISTL |
| **ORF 3** | 107 | 101 | KTHISTLPF |
| **Maxi Peptide Pool ORF 4** | | | |
| **Subpool** | **Index** | **AA Start** | **Peptide Sequence** |
| **ORF 4** | 108 | 5 | VSVNGSSQF |
| **ORF 4** | 109 | 6 | SVNGSSQFF |
| **ORF 4** | 110 | 10 | SSQFFSLTL |
| **ORF 4** | 111 | 11 | SQFFSLTLL |
| **ORF 4** | 112 | 17 | TLLSVAAMI |
| **ORF 4** | 113 | 20 | SVAAMILSV |
| **ORF 4** | 114 | 22 | AAMILSVTF |
| **ORF 4** | 115 | 23 | AMILSVTFV |
| **ORF 4** | 116 | 24 | MILSVTFVA |
| **ORF 4** | 117 | 25 | ILSVTFVAW |
| **ORF 4** | 118 | 28 | VTFVAWLPC |
| **ORF 4** | 119 | 31 | VAWLPCLTV |
| **ORF 4** | 120 | 51 | LLRSGAYPL |
| **ORF 4** | 121 | 55 | GAYPLSRLL |
| **ORF 4** | 122 | 61 | RLLPKSPVI |
| **ORF 4** | 123 | 69 | IEHHGLWVV |
| **ORF 4** | 124 | 76 | VVGPETQTL |
| **HLA-E Peptides** | | | |
| | **Index** | | **Peptide Sequence** |
| | HLA-E_1 | | KIPLKAAQL |
| | HLA-E_2 | | RSPFTNPQL |
| | HLA-E_3 | | TGPSIHPGL |
| | HLA-E_4 | | IQRKKRLLL |
| | HLA-E_5 | | RVPRSVQIV |
| | HLA-E_6 | | RVRERKWLL |
| | HLA-E_7 | | KSSKKKTYL |
| | HLA-E_8 | | LAPLETQAL |
| | HLA-E_9 | | APPEDPVLL |
| | HLA-E_10 | | SGGPRTPQI |
| | HLA-E_11 | | SGAYPLSRL |
| | HLA-E_12 | | ASKKARQLL |
| | HLA-E_13 | | QTEPFSTEL |
| | HLA-E_14 | | LYDNTTKKL |
| | HLA-E_15 | | RIQRKKRLL |
| | HLA-E_16 | | TFTGGSPYL |

| | |
|---|---|
| HLA-E_17 | LINTMPPFL |
| HLA-E_18 | PQIRRLPAL |
| HLA-E_19 | AYNTAITKI |
| HLA-E_20 | LYDEYKRFM |
| HLA-E_21 | LHEKITQYL |
| HLA-E_22 | SGNRVPRSV |
| HLA-E_23 | GMTTDKFTL |
| HLA-E_24 | NRVPRSVQI |
| HLA-E_25 | MPPFLDTQL |
| HLA-E_26 | LLSVAAMIL |
| HLA-E_27 | NGSSQFFSL |
| HLA-E_28 | PTPSAVPTI |
| HLA-E_29 | KGKALHEKI |
| HLA-E_30 | RRACLAQKL |
| HLA-E_31 | NTTQTLAQL |
| HLA-E_32 | KGGGRNIFI |
| HLA-E_33 | ALNKRARWI |
| HLA-E_34 | LWFFRHPEV |
| HLA-E_35 | GNPVSQQVV |
| HLA-E_36 | LRGYVPYSL |
| HLA-E_37 | AWDFRRACL |
| HLA-E_38 | RYRRDTEAL |
| HLA-E_39 | GYVPYSLNF |
| HLA-E_40 | TKHTYRPYL |
| HLA-E_41 | KARQLLTGM |
| HLA-E_42 | RNPCKDTGV |
| HLA-E_43 | YHGGLYSSI |
| HLA-E_44 | KIRVGAPRM |
| HLA-E_45 | YSKTQSKCL |
| HLA-E_46 | PAPPEDPVL |
| HLA-E_47 | PNYNTTQTL |
| HLA-E_48 | RACLAQKLL |
| HLA-E_49 | SSSSDQAPL |
| HLA-E_50 | NTAITKIPL |
| HLA-E_51 | QSDLCDLPL |
| HLA-E_52 | SVYSKTQSK |
| HLA-E_53 | ATHSDDSYL |

Table S2: Overview to TTV-derived Peptide Variants

| Peptide Variant | GenBank (TTV Species)) |
|---|---|
| (A1): SRPGKKHYI | AB008394 (TTV Species 1)<br>AB011486 (TTV Species 1)<br>AB011487 (TTV Species 1)<br>AB011489 (TTV Species 1)<br>AB017610 (TTV Species 1)<br>AB026345 (TTV Species 1)<br>AB026346 (TTV Species 1)<br>AB026347 (TTV Species 1)<br>AB041007 (TTV Species 1)<br>AF116842 (TTV Species 1)<br>AF122913 (TTV Species 1)<br>AF122915 (TTV Species 1)<br>AF129887 (TTV Species 1)<br>AF254410 (TTV Species 1)<br>NC_002076 (TTV Species 1) |
| (A2): NRPGKKHYI | AB011488 (TTV Species 1)<br>AB011490 (TTV Species 1)<br>AB011491 (TTV Species 1)<br>AB011493 (TTV Species 1)<br>AB011494 (TTV Species 1)<br>AB021877 (TTV Species 1) |
| (A3): NRPGRKHYV | AB030486 (TTV Species 1)<br>AF122914 (TTV Species 1)<br>AF122916 (TTV Species 1)<br>AF122920 (TTV Species 1) |
| (A4): TRPGRRHIV | AB030487 (TTV Species 1)<br>AB030489 (TTV Species 1)<br>AF122917 (TTV Species 1)<br>AF122919 (TTV Species 1) |
| (A5): TRPSRRHIV | AB030488 (TTV Species 1) |
| (A6): SIPGKKHYI | AF079173 (TTV Species 1) |
| (A7): NRPGKKHYV | AF122918 (TTV Species 1) |
| (A8): SRQEKKHYI | AF351132 (TTV Species 1) |
| (A9): SRPGRKHVV | AY026466 (TTV Species 1)<br>MH017570 (TTV Species 1)<br>MH017571 (TTV Species 1)<br>MH017572 (TTV Species 1)<br>MH017573 (TTV Species 1)<br>MH017574 (TTV Species 1)<br>MH017576 (TTV Species 1)<br>MH017577 (TTV Species 1)<br>MH017578 (TTV Species 1)<br>MH017579 (TTV Species 1)<br>MH017580 (TTV Species 1)<br>MH017581 (TTV Species 1)<br>MH017582 (TTV Species 1)<br>MH017586 (TTV Species 1)<br>MH017587 (TTV Species 1) |
| (A10): TRPGRKHRI | AB049608 (TTV Species 2) |
| (A11): TRPSRKHRV | AF298585 (TTV Species 3)<br>AJ620218 (TTV Species 3)<br>AJ620219 (TTV Species 3)<br>AJ620220 (TTV Species 3)<br>AJ620221 (TTV Species 3)<br>AJ620222 (TTV Species 3)<br>AJ620223 (TTV Species 3)<br>AJ620224 (TTV Species 3) |

| | |
|---|---|
| | AJ620225 (TTV Species 3)<br>FR751488 (TTV Species 3)<br>MH648901 (TTV Species 3)<br>MH648958 (TTV Species 3)<br>MH649242 (TTV Species 3)<br>MH649258 (TTV Species 3)<br>MT501645 (TTV Species 3) |
| (A12): TRPNKKHKV | JN231329 (TTV Species 3)<br>MH649208 (TTV Species 3) |
| (A13): TRPNRKHKV | KT163904 (TTV Species 3)<br>MH649092 (TTV Species 3)<br>MH649156 (TTV Species 3) |
| (A14): TRPSKKHKV | KT163917 (TTV Species 3) |
| (A15): TRPSKKHYV | NC_014081 (TTV Species 3) |
| (A16): TRPSRRHKI | AB041957 (TTV Species 4) |
| (A17): TRPSRKHVV | AF315076 (TTV Species 5)<br>AF345522 (TTV Species 5) |
| (A18): TRPSKKHYI | AF345523 (TTV Species 5) |
| (A19): TRPRGKKRI | NC_014094 (TTV Species 6) |
| (A20): TKPKGKKTV | AB064604 (TTV Species 9)<br>NC_014075 (TTV Species 9) |
| (A21): TKPKGKSYV | AB064606 (TTV Species 9)<br>AY026465 (TTV Species 9) |
| (A22): TRPGGKPTV | AF345524 (TTV Species 9)<br>AF345525 (TTV Species 9) |
| (A23): TKPKGGSYV | AJ620227 (TTV Species 9)<br>AJ620228 (TTV Species 9)<br>AJ620229 (TTV Species 9) |
| (A24): TRPKGRKAI | AJ620230 (TTV Species 9)<br>AJ620231 (TTV Species 9)<br>AJ620232 (TTV Species 9)<br>AJ620233 (TTV Species 9)<br>AJ620234 (TTV Species 9)<br>AJ620235 (TTV Species 9) |
| (A25): TKPKGPSYI | DQ186999 (TTV Species 9)<br>DQ187000 (TTV Species 9)<br>DQ187001 (TTV Species 9)<br>DQ187002 (TTV Species 9)<br>DQ187003 (TTV Species 9) |
| (A26): TKPRGSGKI | DQ187004 (TTV Species 9)<br>DQ187005 (TTV Species 9)<br>DQ187006 (TTV Species 9)<br>DQ187007 (TTV Species 9) |
| (A27): TKPKGKATV | GU797360 (TTV Species 10) |
| (A28): TKPRGKATV | NC_014076 (TTV Species 10) |
| (A29): TKPKGKKTI | AF315077 (TTV Species 13)<br>AF345526 (TTV Species 13)<br>NC_038339 (TTV Species 13) |
| (A30): TRPRGKKYI | AY823989 (TTV Species 13) |
| (A31): TRPKGRKTV | FR751492 (TTV Species 13)<br>FR751493 (TTV Species 13)<br>FR751494 (TTV Species 13)<br>FR751495 (TTV Species 13)<br>FR751496 (TTV Species 13)<br>FR751497 (TTV Species 13) |
| (A32): TRPGGKKTV | NC_014077 (TTV Species 14) |
| (A33): TFPRGRKYV | AB060593 (TTV Species 15) |

| | |
|---|---|
| (A34): TRPGGKRFV | AF247137 (TTV Species 15) |
| (A35): TRPGGRRFV | AF345521 (TTV Species 15)<br>AX025677 (TTV Species 15)<br>FR751498 (TTV Species 15)<br>FR751499 (TTV Species 15)<br>NC_014091 (TTV Species 15) |
| (A36): TRPGGRRYV | AX025667 (TTV Species 15) |
| (A37): TYPRGRKYV | AX025822 (TTV Species 15) |
| (A38): TFPKGRKYV | AY449524 (TTV Species 15) |
| (A39): TYPRGRKFV | NC_014096 (TTV Species 15) |
| (A40): TRPFGKKAV | AX025718 (TTV Species 18) |
| (A41): TNPRGRQKI | AB025946 (TTV Species 19)<br>AB028669 (TTV Species 19)<br>AB038620 (TTV Species 19)<br>AF345529 (TTV Species 19)<br>AJ620226 (TTV Species 19)<br>DQ186996 (TTV Species 19)<br>DQ186997 (TTV Species 19)<br>DQ186998 (TTV Species 19) |
| (A42): TNPKGRQKI | AB038619 (TTV Species 19) |
| (A43): TRPRGRQKI | AX025761 (TTV Species 19) |
| (A44): TWPKGRSKV | AB060595 (TTV Species 20) |
| (A45): TKPKGREKI | AF345527 (TTV Species 20) |
| (A46): THPKGRAKI | HM224451 (TTV Species 20)<br>NC_038340 (TTV Species 20) |
| (A47): TKPKGKAKI | AB060596 (TTV Species 21) |
| (A48): TKPGGKSRI | AF345528 (TTV Species 21)<br>NC_038341 (TTV Species 21) |
| (A49): TRPGGPQKI | AB049607 (TTV Species 24) |
| (A50): TRPGGRRRV | AF371370 (TTV Species 24)<br>AY823988 (TTV Species 24)<br>DQ186994 (TTV Species 24)<br>DQ186995 (TTV Species 24) |
| (A51): TKPGGRAKI | AX174942 (TTV Species 24)<br>AX174943 (TTV Species 24) |
| (A52): TKPKGRPKI | FR751476 (TTV Species 24)<br>FR751477 (TTV Species 24)<br>FR751478 (TTV Species 24)<br>FR751479 (TTV Species 24)<br>FR751480 (TTV Species 24)<br>FR751481 (TTV Species 24)<br>FR751482 (TTV Species 24)<br>FR751483 (TTV Species 24)<br>FR751485 (TTV Species 24)<br>FR751486 (TTV Species 24)<br>FR751487 (TTV Species 24) |
| (A53): TKPKGKRSI | AB038621 (TTV Species 29)<br>AB038622 (TTV Species 29)<br>AB038623 (TTV Species 29)<br>AB038624 (TTV Species 29) |
| (A54): TKPRGKPYV | AB064595 (TTV Species 29) |
| (A55): TKPRGRKAI | AB064597 (TTV Species 29) |
| (A56): TKPKGKRAI | AB064600 (TTV Species 29) |

| | |
|---|---|
| (A57): TKPRGKRYI | AB064601 (TTV Species 29) |
| (A58): TRPKGKRSV | AB064602 (TTV Species 29) |
| (A59): TKPKGRRSI | AB064603 (TTV Species 29) |
| (A60): SRQGRKHVV | MH017583 (TTV Species 1)<br>MH017584 (TTV Species 1)<br>MH017585 (TTV Species 1) |
| (A61): TKPFGRKRV | KM593802 (TTV Species 15)<br>KM593803 (TTV Species 15) |
| (A62): TKPFGRKKV | AX025838 (TTV Species 18) |
| (A63): TRPRGRKKV | AB050448 (TTV Species 21)<br>AB060592 (TTV Species 21) |
| (A64): KPKGKGTVK | AJ620212 (TTV Species 6)<br>AJ620213 (TTV Species 6)<br>AJ620215 (TTV Species 6)<br>AJ620216 (TTV Species 6)<br>AJ620217 (TTV Species 6) |
| (A65): TRPKGHSTK | AB054648 (TTV Species 7) |
| (A66): TRPKGRIKR | DQ361268 (TTV Species 7) |
| (A67): TKPKGRPVK | LC385662 (TTV Species 7)<br>NC_014080 (TTV Species 7) |
| (A68): TRPRGRATK | NC_014084 (TTV Species 7) |
| (A69): TRPKGKKYK | AX025830 (TTV Species 17) |
| (A70): TRPRGRKRY | AB060597 (TTV Species 24) |
| (A71): TKKGGKKYV | AB064596 (TTV Species 29) |
| (A72): TRPKGKRSK | AB064598 (TTV Species 29) |
| (A73): TKPHGRKYK | AB064599 (TTV Species 29) |
| (B1): MTYAFKWVW | MH017579 (TTV Species 1)<br>MH017580 (TTV Species 1)<br>MH017581 (TTV Species 1)<br>MH017587 (TTV Species 1) |
| (B2): IKYKFDWKW | NC_014081 (TTV Species 3) |
| (B3): SRYKFKFTW | AB041957 (TTV Species 4) |
| (B4): AKYKFRWKW | AF315076 (TTV Species 5)<br>AF345522 (TTV Species 5) |
| (B5): TKYKFHFLF | AJ620212 (TTV Species 6)<br>AJ620213 (TTV Species 6)<br>AJ620215 (TTV Species 6)<br>AJ620216 (TTV Species 6)<br>AJ620217 (TTV Species 6) |
| (B6): SKYNFKFTW | NC_014094 (TTV Species 6) |
| (B7): AKYKFTFLF | AB054648 (TTV Species 7) |
| (B8): MKYKFKFLF | DQ361268 (TTV Species 7) |
| (B9): AKYRFRFLF | LC385662 (TTV Species 7)<br>NC_014080 (TTV Species 7) |
| (B10): AKYKFNFLF | NC_014084 (TTV Species 7) |
| (B11): MKYSFRFTW | AB064604 (TTV Species 9)<br>NC_014075 (TTV Species 9) |

| | |
|---|---|
| (B12): AKYKFTFNF | AB064606 (TTV Species 9)<br>AJ620227 (TTV Species 9)<br>AJ620228 (TTV Species 9)<br>AJ620229 (TTV Species 9)<br>DQ186999 (TTV Species 9)<br>DQ187000 (TTV Species 9)<br>DQ187001 (TTV Species 9)<br>DQ187002 (TTV Species 9)<br>DQ187003 (TTV Species 9)<br>AB064602 (TTV Species 29) |
| (B13): AKYKFRWHF | AF345524 (TTV Species 9) |
| (B14): TKYKFTFNF | AY026465 (TTV Species 9) |
| (B15): IKYKAKFIF | DQ187004 (TTV Species 9)<br>DQ187005 (TTV Species 9)<br>DQ187006 (TTV Species 9)<br>DQ187007 (TTV Species 9) |
| (B16): AKYKFKFLF | GU797360 (TTV Species 10)<br>NC_014076 (TTV Species 10) |
| (B17): AKYRFTFYW | AF315077 (TTV Species 13)<br>AF345526 (TTV Species 13)<br>NC_038339 (TTV Species 13) |
| (B18): AGYTFRFKW | AY823989 (TTV Species 13) |
| (B19): AKYRFSFIW | FR751492 (TTV Species 13)<br>FR751493 (TTV Species 13)<br>FR751494 (TTV Species 13)<br>FR751495 (TTV Species 13)<br>FR751496 (TTV Species 13)<br>FR751497 (TTV Species 13) |
| (B20): SPYTFRFNW | AB060593 (TTV Species 15) |
| (B21): AMYSFKFLF | AX025667 (TTV Species 15) |
| (B22): VYYNFIFNW | AX025677 (TTV Species 15)<br>FR751499 (TTV Species 15) |
| (B23): AGYSFRFNW | AX025730 (TTV Species 15)<br>NC_014096 (TTV Species 15) |
| (B24): ASYTFRFNW | AX025822 (TTV Species 15)<br>AY449524 (TTV Species 15) |
| (B25): AEYKFRFNW | KM593802 (TTV Species 15)<br>KM593803 (TTV Species 15) |
| (B26): MAYNFKFNW | AX025830 (TTV Species 17) |
| (B27): AKYKFYFNW | AX025718 (TTV Species 18) |
| (B28): AGYKFRFNW | AX025838 (TTV Species 18) |
| (B29): AKYKFYFTW | AB025946 (TTV Species 19)<br>AB028669 (TTV Species 19)<br>AX025761 (TTV Species 19) |
| (B30): MKYKFRFTW | AJ620226 (TTV Species 19) |
| (B31): AKYKFKFKW | AB060595 (TTV Species 20) |
| (B32): AKYRFDFKW | AF345527 (TTV Species 20) |
| (B33): AKYKFRFKW | HM224451 (TTV Species 20)<br>NC_038340 (TTV Species 20) |

| | |
|---|---|
| (B34): VEYKFKFLW | AB050448 (TTV Species 21)<br>AB060592 (TTV Species 21) |
| (B35): AKYKFSFLW | AB060596 (TTV Species 21) |
| (B36): TTYKFKFLW | AF345528 (TTV Species 21)<br>NC_038341 (TTV Species 21) |
| (B37): AAYSFKFLW | AB049607 (TTV Species 24) |
| (B38): ARYKFRFKW | AB060597 (TTV Species 24) |
| (B39): AGYKFKFTW | AF371370 (TTV Species 24) |
| (B40): AKYKFDFMW | AX174942 (TTV Species 24)<br>AX174943 (TTV Species 24) |
| (B41): AGYKFRFSW | AY823988 (TTV Species 24) |
| (B42): AGYKFKFSW | DQ003341 (TTV Species 24)<br>DQ003342 (TTV Species 24)<br>DQ003343 (TTV Species 24)<br>DQ003344 (TTV Species 24)<br>DQ186994 (TTV Species 24)<br>DQ186995 (TTV Species 24) |
| (B43): AKYTFDFMW | FR751476 (TTV Species 24)<br>FR751477 (TTV Species 24)<br>FR751478 (TTV Species 24)<br>FR751479 (TTV Species 24)<br>FR751480 (TTV Species 24)<br>FR751481 (TTV Species 24)<br>FR751482 (TTV Species 24)<br>FR751483 (TTV Species 24)<br>FR751485 (TTV Species 24)<br>FR751486 (TTV Species 24)<br>FR751487 (TTV Species 24) |
| (B44): MKYAFKWVW | AY026466 (TTV Species 1)<br>MH017570 (TTV Species 1)<br>MH017571 (TTV Species 1)<br>MH017572 (TTV Species 1)<br>MH017573 (TTV Species 1)<br>MH017574 (TTV Species 1)<br>MH017575 (TTV Species 1)<br>MH017576 (TTV Species 1)<br>MH017577 (TTV Species 1)<br>MH017578 (TTV Species 1)<br>MH017582 (TTV Species 1)<br>MH017583 (TTV Species 1)<br>MH017586 (TTV Species 1) |
| (B45): TKYAFKWVW | MH017584 (TTV Species 1)<br>MH017585 (TTV Species 1) |
| (B46): MKYRFHWKW | AF298585 (TTV Species 3)<br>AJ620218 (TTV Species 3)<br>AJ620219 (TTV Species 3)<br>AJ620220 (TTV Species 3)<br>AJ620221 (TTV Species 3)<br>AJ620222 (TTV Species 3)<br>AJ620223 (TTV Species 3)<br>AJ620224 (TTV Species 3)<br>AJ620225 (TTV Species 3) |

| | |
|---|---|
| | FR751488 (TTV Species 3)<br>MH648901 (TTV Species 3)<br>MH648958 (TTV Species 3)<br>MH649242 (TTV Species 3)<br>MT501645 (TTV Species 3) |
| (B47): IKYNFRWKW | KT163917 (TTV Species 3)<br>AF345523 (TTV Species 5) |
| (B48): LYYNFIFNW | AF247137 (TTV Species 15)<br>FR751498 (TTV Species 15)<br>NC_014091 (TTV Species 15) |
| (B49): MGYKMDWKW | AB064603 (TTV Species 29) |
| (B50): IKYRFKWVW | AB030486 (TTV Species 1)<br>AF122920 (TTV Species 1) |
| (B51): LKYKFHWIW | AB030489 (TTV Species 1) |
| (B52): IGYKFLWRW | AB064596 (TTV Species 29) |
| (B53): VKYKFKWIW | AB049608 (TTV Species 2) |
| (B54): MKYVFKWKW | KT163904 (TTV Species 3)<br>MH649156 (TTV Species 3) |
| (B55): LKYRFHWKW | MH649258 (TTV Species 3) |
| (B56): AYYNFIFDW | AF345521 (TTV Species 15) |
| (B57): CKYKFYFTW | AB038619 (TTV Species 19)<br>AB038620 (TTV Species 19)<br>AF345529 (TTV Species 19)<br>DQ186996 (TTV Species 19)<br>DQ186997 (TTV Species 19)<br>DQ186998 (TTV Species 19) |
| (B58): MGYKMDWLW | AB038621 (TTV Species 29)<br>AB038622 (TTV Species 29)<br>AB038623 (TTV Species 29)<br>AB038624 (TTV Species 29) |
| (B59): MKYRFKWIW | AB008394 (TTV Species 1)<br>AB011486 (TTV Species 1)<br>AB011487 (TTV Species 1)<br>AB011489 (TTV Species 1)<br>AB017610 (TTV Species 1)<br>AB026345 (TTV Species 1)<br>AB026346 (TTV Species 1)<br>AB026347 (TTV Species 1)<br>AB041007 (TTV Species 1)<br>AF079173 (TTV Species 1)<br>AF116842 (TTV Species 1)<br>AF122913 (TTV Species 1)<br>AF122915 (TTV Species 1)<br>AF351132 (TTV Species 1)<br>NC_002076 (TTV Species 1) |
| (B60): IKYRFKWIW | AB011488 (TTV Species 1)<br>AB011490 (TTV Species 1)<br>AB011491 (TTV Species 1)<br>AB011493 (TTV Species 1)<br>AB011494 (TTV Species 1)<br>AB021877 (TTV Species 1) |

| | |
|---|---|
| | AF122914 (TTV Species 1)<br>AF129887 (TTV Species 1)<br>AF254410 (TTV Species 1) |
| (B61): LKYRFHWIW | AB030487 (TTV Species 1)<br>AB030488 (TTV Species 1)<br>AF122917 (TTV Species 1)<br>AF122919 (TTV Species 1) |
| (B62): LKYRFKWVW | AF122916 (TTV Species 1)<br>AF122918 (TTV Species 1) |
| (B63): MKYVFNWKW | JN231329 (TTV Species 3)<br>MH649092 (TTV Species 3) |
| (B64): LKYIFRWKW | MH649208 (TTV Species 3) |
| (B65): MKYCFNFNW | AF345525 (TTV Species 9)<br>AJ620230 (TTV Species 9)<br>AJ620231 (TTV Species 9)<br>AJ620232 (TTV Species 9)<br>AJ620233 (TTV Species 9)<br>AJ620234 (TTV Species 9)<br>AJ620235 (TTV Species 9) |
| (B66): CKYKFRFKW | NC_014077 (TTV Species 14) |
| (C1): TTDKFTLRI | AB008394 (TTV Species 1)<br>AB011486 (TTV Species 1)<br>AB011487 (TTV Species 1)<br>AB011489 (TTV Species 1)<br>AB017610 (TTV Species 1)<br>AB026345 (TTV Species 1)<br>AB026346 (TTV Species 1)<br>AB026347 (TTV Species 1)<br>AB030486 (TTV Species 1)<br>AB041007 (TTV Species 1)<br>AF079173 (TTV Species 1)<br>AF116842 (TTV Species 1)<br>AF122913 (TTV Species 1)<br>AF122915 (TTV Species 1)<br>AF122916 (TTV Species 1)<br>AF122920 (TTV Species 1)<br>AF129887 (TTV Species 1)<br>AF254410 (TTV Species 1)<br>AF351132 (TTV Species 1)<br>AY026466 (TTV Species 1)<br>MH017571 (TTV Species 1)<br>MH017579 (TTV Species 1)<br>MH017586 (TTV Species 1)<br>MH017587 (TTV Species 1)<br>NC_002076 (TTV Species 1) |

| | |
|---|---|
| (C2): TTDKFSLRI | AB011488 (TTV Species 1)<br>AB011490 (TTV Species 1)<br>AB011491 (TTV Species 1)<br>AB011493 (TTV Species 1)<br>AB011494 (TTV Species 1)<br>AB021877 (TTV Species 1)<br>AF122914 (TTV Species 1)<br>MH017572 (TTV Species 1) |
| (C3): ATQKFTLRI | AB030487 (TTV Species 1)<br>AB030488 (TTV Species 1)<br>AB030489 (TTV Species 1)<br>AF122919 (TTV Species 1)<br>FR751483 (TTV Species 24) |
| (C4): ATQKFTPRI | AF122917 (TTV Species 1) |
| (C5): TADKFTLRI | AF122918 (TTV Species 1) |
| (C6): TTDKFTPRI | MH017570 (TTV Species 1) |
| (C7): TTGKFSLRI | MH017573 (TTV Species 1)<br>MH017574 (TTV Species 1)<br>MH017575 (TTV Species 1)<br>MH017576 (TTV Species 1)<br>MH017577 (TTV Species 1)<br>MH017578 (TTV Species 1)<br>MH017582 (TTV Species 1) |
| (C8): TTDKFTLKI | MH017580 (TTV Species 1)<br>MH017581 (TTV Species 1) |
| (C9): TIDKFTLRI | MH017583 (TTV Species 1)<br>MH017584 (TTV Species 1)<br>MH017585 (TTV Species 1) |
| (C10): TTTKFTLRI | AF298585 (TTV Species 3)<br>AJ620218 (TTV Species 3)<br>AJ620219 (TTV Species 3)<br>AJ620220 (TTV Species 3)<br>AJ620221 (TTV Species 3)<br>AJ620222 (TTV Species 3)<br>AJ620223 (TTV Species 3)<br>AJ620224 (TTV Species 3)<br>AJ620225 (TTV Species 3)<br>FR751488 (TTV Species 3)<br>JN231329 (TTV Species 3)<br>KT163904 (TTV Species 3)<br>KT163917 (TTV Species 3)<br>MH648901 (TTV Species 3)<br>MH648958 (TTV Species 3)<br>MH649092 (TTV Species 3)<br>MH649156 (TTV Species 3)<br>MH649208 (TTV Species 3)<br>MH649242 (TTV Species 3)<br>MH649258 (TTV Species 3)<br>MT501645 (TTV Species 3)<br>NC_014081 (TTV Species 3) |
| (C11): TTMQFSLRI | AB041957 (TTV Species 4) |

| | |
|---|---|
| (C12): CTAQYTLRI | AF315076 (TTV Species 5)<br>AF345522 (TTV Species 5) |
| (C13): CTTKFTLRI | AF345523 (TTV Species 5) |
| (C14): SSMRFNMRI | AJ620212 (TTV Species 6)<br>AJ620213 (TTV Species 6)<br>AJ620215 (TTV Species 6)<br>AJ620216 (TTV Species 6)<br>AJ620217 (TTV Species 6) |
| (C15): STMQFNLSI | AB054648 (TTV Species 7) |
| (C16): STMRFSLKV | AB064606 (TTV Species 9)<br>AJ620227 (TTV Species 9)<br>AJ620228 (TTV Species 9)<br>AJ620229 (TTV Species 9)<br>AY026465 (TTV Species 9)<br>DQ186999 (TTV Species 9)<br>DQ187000 (TTV Species 9)<br>DQ187001 (TTV Species 9)<br>DQ187002 (TTV Species 9)<br>DQ187003 (TTV Species 9) |
| (C17): STARLSLK | AF345524 (TTV Species 9) |
| (C18): STSRWSLKV | AF345525 (TTV Species 9) |
| (C19): STMRFSLYI | AJ620230 (TTV Species 9)<br>AJ620231 (TTV Species 9)<br>AJ620232 (TTV Species 9)<br>AJ620233 (TTV Species 9)<br>AJ620234 (TTV Species 9)<br>AJ620235 (TTV Species 9) |
| (C20): GTARMSLKV) | DQ187004 (TTV Species 9)<br>DQ187006 (TTV Species 9)<br>DQ187007 (TTV Species 9 |
| (C21): GTAGMSLKV | DQ187005 (TTV Species 9) |
| (C22): STMRFSLQV | NC_014075 (TTV Species 9) |
| (C23): ATTRWSLKV | GU797360 (TTV Species 10)<br>NC_014076 (TTV Species 10) |
| (C24): ATETFSLRV | AF315077 (TTV Species 13)<br>AF345526 (TTV Species 13)<br>NC_038339 (TTV Species 13) |
| (C25): GTETFSLRV | AY823989 (TTV Species 13) |
| (C26): ATETFSLKV | FR751492 (TTV Species 13)<br>FR751493 (TTV Species 13)<br>FR751494 (TTV Species 13)<br>FR751495 (TTV Species 13)<br>FR751496 (TTV Species 13)<br>FR751497 (TTV Species 13) |
| (C27): SLTTFNLRA | NC_014077 (TTV Species 14) |
| (C28): STVTFSLYV | AB060593 (TTV Species 15)<br>AX025822 (TTV Species 15)<br>AY449524 (TTV Species 15) |

| | |
|---|---|
| (C29): STVTFSLKA | AF247137 (TTV Species 15)<br>NC_014091 (TTV Species 15) |
| (C30): STTTFSLRG | AF345521 (TTV Species 15) |
| (C31): STITFSLLV | AX025667 (TTV Species 15) |
| (C32): STTTFSLRA | AX025677 (TTV Species 15)<br>FR751498 (TTV Species 15)<br>FR751499 (TTV Species 15) |
| (C33): STVTFSLFV | AX025730 (TTV Species 15)<br>NC_014096 (TTV Species 15) |
| (C34): STTSFSLKV | AB025946 (TTV Species 19)<br>AB028669 (TTV Species 19)<br>AB038619 (TTV Species 19)<br>AB038620 (TTV Species 19)<br>AF345529 (TTV Species 19)<br>DQ186996 (TTV Species 19)<br>DQ186997 (TTV Species 19)<br>DQ186998 (TTV Species 19) |
| (C35): STTSFNLKV | AJ620226 (TTV Species 19) |
| (C36): STTSFNLRV | AX025761 (TTV Species 19) |
| (C37): STTSYSLKL | AB060595 (TTV Species 20) |
| (C38): STVSFSLKV | AF345527 (TTV Species 20) |
| (C39): STTTFTLKV | HM224451 (TTV Species 20)<br>NC_038340 (TTV Species 20) |
| (C40): STTTWSLKV | AB050448 (TTV Species 21)<br>AB060592 (TTV Species 21)<br>AB049607 (TTV Species 24)<br>AB060597 (TTV Species 24)<br>AX174942 (TTV Species 24)<br>AX174943 (TTV Species 24)<br>FR751476 (TTV Species 24)<br>FR751477 (TTV Species 24)<br>FR751478 (TTV Species 24)<br>FR751479 (TTV Species 24)<br>FR751480 (TTV Species 24)<br>FR751481 (TTV Species 24)<br>FR751482 (TTV Species 24)<br>FR751485 (TTV Species 24)<br>FR751486 (TTV Species 24)<br>FR751487 (TTV Species 24) |
| (C41): STTTWSLRV | AF345528 (TTV Species 21)<br>NC_038341 (TTV Species 21) |
| (C42): STTTWNLRV | AF371370 (TTV Species 24)<br>AY823988 (TTV Species 24)<br>DQ003341 (TTV Species 24)<br>DQ003342 (TTV Species 24)<br>DQ003343 (TTV Species 24)<br>DQ003344 (TTV Species 24)<br>DQ186994 (TTV Species 24)<br>DQ186995 (TTV Species 24) |
| (C43): TNLTFSLEG | AB038621 (TTV Species 29)<br>AB038623 (TTV Species 29) |

| | |
|---|---|
| | AB038624 (TTV Species 29) |
| (C44): TNLSFSLEG | AB038622 (TTV Species 29) |
| (C45): THITWCLEA | AB064595 (TTV Species 29)<br>AB064596 (TTV Species 29) |
| (C46): TNMTFSLEA | AB064597 (TTV Species 29) |
| (C47): TNLTFTLAG | AB064598 (TTV Species 29) |
| (C48): TNITFTLAA | AB064601 (TTV Species 29) |
| (C49): TNITITLGG | AB064602 (TTV Species 29) |
| (C50): VNVTFSLEA | AB064603 (TTV Species 29) |
| (C51): TATQFTLRI | AB049608 (TTV Species 2) |
| (C52): STITISLQV | AX025830 (TTV Species 17) |
| (C53): STTTWSLKL | AB060596 (TTV Species 21) |
| (C54): TTTMFNLKV | NC_014094 (TTV Species 6) |
| (C55): STMLFTMQN | DQ361268 (TTV Species 7) |
| (C56): STLMFNLRI | LC385662 (TTV Species 7)<br>NC_014080 (TTV Species 7) |
| (C57): STMQFTLET | NC_014084 (TTV Species 7) |
| (C58): ATFRFSLQV | AB064604 (TTV Species 9) |
| (C59): STVTFSLQV | KM593802 (TTV Species 15)<br>KM593803 (TTV Species 15)<br>AX025838 (TTV Species 18) |
| (C60): STVTFSLKV | AX025718 (TTV Species 18) |
| (C61): TNLTFNLAC | AB064599 (TTV Species 29) |
| (C62): TNLTFTLEG | AB064600 (TTV Species 29) |
| (D1): LRDTPFYPW | AB008394 (TTV Species 1)<br>AB017610 (TTV Species 1)<br>AB026345 (TTV Species 1)<br>AF254410 (TTV Species 1)<br>AF351132 (TTV Species 1) |
| (D2): LRDPPFYPW | AB026346 (TTV Species 1)<br>AB026347 (TTV Species 1)<br>AF122914 (TTV Species 1) |
| (D3): LRDTPYYPW | AB041007 (TTV Species 1)<br>AF122913 (TTV Species 1)<br>NC_002076 (TTV Species 1) |
| (D4): LRSTPHYPW | AF122916 (TTV Species 1) |
| (D5): LRDNPFYPW | AF122917 (TTV Species 1) |
| (D6): LRDKPFYPW | AF122919 (TTV Species 1) |
| (D7): LNDIPFYHW | MH017583 (TTV Species 1)<br>MH017584 (TTV Species 1)<br>MH017585 (TTV Species 1) |
| (D8): HTDTPFYPW | AF298585 (TTV Species 3)<br>KT163904 (TTV Species 3)<br>MH648958 (TTV Species 3)<br>MH649092 (TTV Species 3)<br>MH649156 (TTV Species 3)<br>MH649208 (TTV Species 3) |

| | |
|---|---|
| | FR751492 (TTV Species 13)<br>FR751493 (TTV Species 13)<br>FR751494 (TTV Species 13)<br>FR751495 (TTV Species 13)<br>FR751496 (TTV Species 13)<br>FR751497 (TTV Species 13) |
| (D9): HTDIPFYPW | AJ620218 (TTV Species 3)<br>AJ620219 (TTV Species 3)<br>AJ620220 (TTV Species 3)<br>AJ620221 (TTV Species 3)<br>AJ620222 (TTV Species 3)<br>AJ620224 (TTV Species 3)<br>AJ620225 (TTV Species 3)<br>MH648901 (TTV Species 3)<br>MH649242 (TTV Species 3)<br>MH649258 (TTV Species 3)<br>MT501645 (TTV Species 3) |
| (D10): LTDPPFYPY | AJ620223 (TTV Species 3)<br>AB064604 (TTV Species 9)<br>NC_014075 (TTV Species 9)<br>GU797360 (TTV Species 10)<br>NC_014076 (TTV Species 10) |
| (D11): HSDLPFYPW | FR751488 (TTV Species 3) |
| (D12): HTDTPYYPW | JN231329 (TTV Species 3) |
| (D13): YTDIPTYTW | NC_014081 (TTV Species 3) |
| (D14): LTKPPFYPW | AF345523 (TTV Species 5) |
| (D15): NDTPFYPYI | AJ620216 (TTV Species 6) |
| (D16): LTDPPFYPW | NC_014094 (TTV Species 6)<br>AB060593 (TTV Species 15)<br>NC_014096 (TTV Species 15) |
| (D17): LSRPPFYPW | AB054648 (TTV Species 7) |
| (D18): LSDTPFYPW | DQ361268 (TTV Species 7)<br>AF345527 (TTV Species 20) |
| (D19): LTDTPYYPW | LC385662 (TTV Species 7)<br>NC_014080 (TTV Species 7) |
| (D20): LSSPPFYPW | NC_014084 (TTV Species 7) |
| (D21): LTDFPYYPF | DQ187004 (TTV Species 9)<br>DQ187005 (TTV Species 9)<br>DQ187006 (TTV Species 9)<br>DQ187007 (TTV Species 9) |
| (D22): LSDTPTYPW | AF315077 (TTV Species 13)<br>AF345526 (TTV Species 13)<br>NC_038339 (TTV Species 13) |
| (D23): LSRPPHYPW | AY823989 (TTV Species 13) |
| (D24): YTDTPYYPW | AF247137 (TTV Species 15)<br>AF345528 (TTV Species 21)<br>NC_038341 (TTV Species 21) |
| (D25): LTDKPYYPW | AX025667 (TTV Species 15) |
| (D26): LQDTPFYPW | FR751498 (TTV Species 15)<br>FR751499 (TTV Species 15) |

| | |
|---|---|
| (D27): LSDKPIYPW | KM593802 (TTV Species 15)<br>KM593803 (TTV Species 15)<br>AX025718 (TTV Species 18)<br>AX025838 (TTV Species 18)<br>FR751476 (TTV Species 24) |
| (D28): YSDTPTYPW | AJ620226 (TTV Species 19) |
| (D29): LSDSPYYPW | AX025761 (TTV Species 19) |
| (D30): LSDNPFYPW | AB060595 (TTV Species 20) |
| (D31): LSDSPFYPW | HM224451 (TTV Species 20) |
| (D32): LSNSPFYPW | NC_038340 (TTV Species 20) |
| (D33): HTDPPFYPW | AB050448 (TTV Species 21) |
| (D34): LKDFYYTQM | AB060592 (TTV Species 21) |
| (D35): YTDTPTYPW | AB060596 (TTV Species 21) |
| (D36): HLDTPTYPW | AB060597 (TTV Species 24) |
| (D37): ATDMPTYPW | AF371370 (TTV Species 24) |
| (D38): LKDIYYNTM | AX174942 (TTV Species 24) |
| (D39): HTDTPTYPW | AY823988 (TTV Species 24)<br>DQ003341 (TTV Species 24)<br>DQ003342 (TTV Species 24)<br>DQ003343 (TTV Species 24)<br>DQ003344 (TTV Species 24)<br>DQ186994 (TTV Species 24)<br>DQ186995 (TTV Species 24) |
| (D40): LNDTPFYPW | MH017571 (TTV Species 1)<br>MH017572 (TTV Species 1)<br>MH017573 (TTV Species 1)<br>MH017574 (TTV Species 1)<br>MH017576 (TTV Species 1)<br>MH017577 (TTV Species 1)<br>MH017586 (TTV Species 1)<br>MH017587 (TTV Species 1) |
| (D41): YSDTPYYPW | FR751478 (TTV Species 24)<br>FR751479 (TTV Species 24)<br>FR751480 (TTV Species 24)<br>FR751481 (TTV Species 24)<br>FR751482 (TTV Species 24)<br>FR751483 (TTV Species 24)<br>FR751485 (TTV Species 24)<br>FR751486 (TTV Species 24)<br>FR751487 (TTV Species 24) |
| (D42): PLDLPHYPW | AB064596 (TTV Species 29) |
| (D43): LRSTPFYPW | AF122918 (TTV Species 1)<br>AF122920 (TTV Species 1) |
| (D44): LRDIPFYPW | AF129887 (TTV Species 1) |
| (D45): LNSPPFYPW | AF315076 (TTV Species 5)<br>AF345522 (TTV Species 5) |
| (D46): LNDKPFYPW | NC_014077 (TTV Species 14) |
| (D47): PSDPPYYPW | AB038624 (TTV Species 29) |
| (D48): PGDPPFYPW | AB064600 (TTV Species 29) |

| | |
|---|---|
| (D49): PTDPPFYPW | AB064601 (TTV Species 29) |
| (D50): LNDTHFYPW | MH017570 (TTV Species 1) |
| (D51): YSDTPHYPW | FR751477 (TTV Species 24) |
| (D52): LNDTPFSPW | MH017578 (TTV Species 1) |
| (D53): LKDTPYYPY | AF345524 (TTV Species 9)<br>AF345525 (TTV Species 9)<br>AJ620230 (TTV Species 9)<br>AJ620231 (TTV Species 9)<br>AJ620232 (TTV Species 9)<br>AJ620233 (TTV Species 9)<br>AJ620234 (TTV Species 9)<br>AJ620235 (TTV Species 9) |
| (D54): LKDNPFYPW | AX025822 (TTV Species 15) |
| (D55): LKDSPFYPW | AY449524 (TTV Species 15) |
| (D56): LTRPPCYPW | AX025830 (TTV Species 17) |
| (D57): PSDPPHYPW | AB038622 (TTV Species 29)<br>AB064598 (TTV Species 29) |
| (D58): LNDIPFYPW | AF079173 (TTV Species 1)<br>MH017575 (TTV Species 1)<br>MH017579 (TTV Species 1)<br>MH017580 (TTV Species 1)<br>MH017581 (TTV Species 1) |
| (D59): LRETPFYPW | AF116842 (TTV Species 1) |
| (D60): LNATPFYPW | MH017582 (TTV Species 1) |
| (D61): HTDIPFYPD | KT163917 (TTV Species 3) |
| (D62): PLDTPFYPW | AB041957 (TTV Species 4) |
| (D63): LNDTPFYPY | AJ620212 (TTV Species 6)<br>AJ620213 (TTV Species 6)<br>AJ620215 (TTV Species 6)<br>AJ620217 (TTV Species 6) |
| (D64): LTDTPFYTH | AB064606 (TTV Species 9)<br>AJ620227 (TTV Species 9) |
| (D65): LKDTPFYPW | AF345521 (TTV Species 15)<br>AX025677 (TTV Species 15)<br>NC_014091 (TTV Species 15) |
| (D66): LKDRPFYPW | AX025730 (TTV Species 15) |
| (D67): LSSPPFYPS | AB028669 (TTV Species 19)<br>AB038620 (TTV Species 19)<br>DQ186996 (TTV Species 19)<br>DQ186997 (TTV Species 19)<br>DQ186998 (TTV Species 19) |
| (D68): LSSPPFYPR | AF345529 (TTV Species 19) |
| (D69): PSDLPHYPW | AB038621 (TTV Species 29)<br>AB064603 (TTV Species 29) |
| (D70): PSDLPTYPW | AB064595 (TTV Species 29) |
| (D71): PSDTPHYPW | AB064597 (TTV Species 29) |
| (D72): PSDNPTYPW | AB064599 (TTV Species 29) |
| (E1): TGNGTVLYL | AB011493 (TTV Species 1) |

| | |
|---|---|
| (E2): TGNGTQVYL | AB008394 (TTV Species 1)<br>AB017610 (TTV Species 1)<br>AB026345 (TTV Species 1)<br>AB026346 (TTV Species 1)<br>AB026347 (TTV Species 1)<br>AB041007 (TTV Species 1)<br>AF079173 (TTV Species 1)<br>AF116842 (TTV Species 1)<br>AF122913 (TTV Species 1)<br>AF122915 (TTV Species 1)<br>AF351132 (TTV Species 1)<br>NC_002076 (TTV Species 1) |
| (E3): NITGSTVAF | AB030486 (TTV Species 1) |
| (E4): SENGSSQLF | AB030487 (TTV Species 1)<br>AB030488 (TTV Species 1) |
| (E5): TGNGTLLYL | AF122914 (TTV Species 1) |
| (E6): NVTGSTVAF | AF122916 (TTV Species 1)<br>AF122918 (TTV Species 1)<br>AF122920 (TTV Species 1) |
| (E7): SENGSSPLF | AF122919 (TTV Species 1) |
| (E8): RDNGTQVYL | AF129887 (TTV Species 1) |
| (E9): SVNGSSQFF | AY026466 (TTV Species 1)<br>MH017570 (TTV Species 1)<br>MH017571 (TTV Species 1)<br>MH017572 (TTV Species 1)<br>MH017573 (TTV Species 1)<br>MH017574 (TTV Species 1)<br>MH017575 (TTV Species 1)<br>MH017576 (TTV Species 1)<br>MH017582 (TTV Species 1)<br>MH017586 (TTV Species 1)<br>MH017587 (TTV Species 1) |
| (E10): SANGSSQFF | MH017577 (TTV Species 1) |
| (E11): SVNGSSRFF | MH017579 (TTV Species 1)<br>MH017580 (TTV Species 1)<br>MH017581 (TTV Species 1)<br>MH017583 (TTV Species 1)<br>MH017584 (TTV Species 1)<br>MH017585 (TTV Species 1) |
| (E12): KGSGTSHAL | KT163917 (TTV Species 3) |
| (E13): RDSGTSPAF | MH649208 (TTV Species 3) |
| (E14): SSNGSSPPL | NC_014091 (TTV Species 15) |
| (E15): NKTGLSPAL | NC_014096 (TTV Species 15) |
| (E16): SENGSSQLS | AB030489 (TTV Species 1) |
| (E17): SENGFSQLS | AF122917 (TTV Species 1) |
| (E18): SANGSSQFS | MH017578 (TTV Species 1) |
| (E19): SVSSSQESK | AF315076 (TTV Species 5) |
| (E20): SYVGTAQFE | AF345526 (TTV Species 13)<br>NC_038339 (TTV Species 13) |

| | |
|---|---|
| (E21): SGSGSSAAT | AY823989 (TTV Species 13) |
| (E22): SLDGTRRFE | FR751492 (TTV Species 13)<br>FR751493 (TTV Species 13)<br>FR751494 (TTV Species 13)<br>FR751496 (TTV Species 13)<br>FR751497 (TTV Species 13) |
| (F1): DVPQKAAKL | AB011488 (TTV Species 1) |
| (F2): GLPIQAAQL | AB011489 (TTV Species 1) |
| (F3): NVPEKAAQL | AB011490 (TTV Species 1) |
| (F4): NVPQKAAEL | AB011491 (TTV Species 1)<br>AB011494 (TTV Species 1)<br>AB021877 (TTV Species 1) |
| (F5): DIPLKAAEL | AB030489 (TTV Species 1) |
| (F6): TIPEKASKL | AF122916 (TTV Species 1) |
| (F7): DLPIKAATL | AF351132 (TTV Species 1) |
| (F8): RIPLKAAQP | MH017570 (TTV Species 1) |
| (F9): KIPLKAAQL | MH017571 (TTV Species 1)<br>MH017572 (TTV Species 1)<br>MH017573 (TTV Species 1)<br>MH017574 (TTV Species 1)<br>MH017575 (TTV Species 1)<br>MH017576 (TTV Species 1)<br>MH017577 (TTV Species 1)<br>MH017578 (TTV Species 1)<br>MH017579 (TTV Species 1)<br>MH017580 (TTV Species 1)<br>MH017581 (TTV Species 1)<br>MH017582 (TTV Species 1)<br>MH017585 (TTV Species 1)<br>MH017586 (TTV Species 1) |
| (F10): KIPFQAAQL | MH017583 (TTV Species 1) |
| (F11): NLPKQAATL | NC_002076 (TTV Species 1) |
| (F12): LAQITRNRF | AJ620221 (TTV Species 3) |
| (F13): NFKKNTDNL | AJ620216 (TTV Species 6) |
| (F14): SFKTNAENL | NC_014094 (TTV Species 6)<br>NC_014084 (TTV Species 7) |
| (F15): AMEENQSNM | DQ361268 (TTV Species 7) |
| (F16): AMKDNATKM | LC385662 (TTV Species 7)<br>NC_014080 (TTV Species 7) |
| (F17): TNPRGRQKI | AB028669 (TTV Species 19) |
| (F18): KLHKIREQY | AJ620226 (TTV Species 19) |
| (F19): KLRKIADWF | AB050448 (TTV Species 21) |
| (F20): TMQNANKWF | AB060597 (TTV Species 24) |
| (F21): KPNNLAVNI | AX174943 (TTV Species 24) |
| (F22): QLIQKEYSY | AB038623 (TTV Species 29) |
| (F23): LVREEYQQL | AB064599 (TTV Species 29) |
| (F24): KIPLKAAQP | MH017584 (TTV Species 1) |

| | |
|---|---|
| (F25): LHKLRQAYF | AB038619 (TTV Species 19) |
| (F26): KLAEKTRQR | AJ620220 (TTV Species 3)<br>AJ620225 (TTV Species 3) |
| (F27): NIETKLYKI | AY823989 (TTV Species 13) |
| (F28): QIGSNANKW | DQ003341 (TTV Species 24)<br>DQ003342 (TTV Species 24)<br>DQ003343 (TTV Species 24)<br>DQ003344 (TTV Species 24) |
| (F29): KDLPTKAAE | AB011486 (TTV Species 1) |
| (F30): KELPKKAAE | AB011487 (TTV Species 1)<br>AF079173 (TTV Species 1)<br>AF129887 (TTV Species 1) |
| (F31): KDVPKKAAE | AB011493 (TTV Species 1)<br>AB008394 (TTV Species 1)<br>AB017610 (TTV Species 1)<br>AF116842 (TTV Species 1) |
| (F33): KDLPKKAAE | AB026345 (TTV Species 1) |
| (F34): SKLPKQAAE | AB026346 (TTV Species 1) |
| (F35): SQLPKKAAE | AB026347 (TTV Species 1) |
| (F36): TEVPPKAAE | AB030486 (TTV Species 1) |
| (F37): TKVPLAAAK | AB030487 (TTV Species 1)<br>AF122919 (TTV Species 1) |
| (F38): KNVPLKAAE | AB030488 (TTV Species 1) |
| (F39): KNLPKQAAT | AB041007 (TTV Species 1) |
| (F40): KDVAKKAAE | AF122913 (TTV Species 1) |
| (F41): KDVPQKAAQ | AF122914 (TTV Species 1) |
| (F42): TEVPIKAAT | AF122915 (TTV Species 1) |
| (F43): TEVPLAAAK | AF122917 (TTV Species 1) |
| (F44): KKIPEEASK | AF122918 (TTV Species 1) |
| (F45): KDIPEKASK | AF122920 (TTV Species 1) |
| (F46): KNLPKQAAE | AF254410 (TTV Species 1) |
| (F47): TNVPLQAAQ | AY026466 (TTV Species 1) |
| (F48): KIRLKAAQP | MH017587 (TTV Species 1) |
| (F49): QVRKDTGDK | AB049608 (TTV Species 2) |
| (F50): KELAEKTRS | AF298585 (TTV Species 3) |
| (F51): QLAEKTRKR | AJ620218 (TTV Species 3) |
| (F52): DLAEKTRNR | AJ620219 (TTV Species 3) |
| (F53): ELAQITRNR | AJ620222 (TTV Species 3) |
| (F54): QLAQITRNR | AJ620223 (TTV Species 3) |
| (F55): KLAEVTRNR | AJ620224 (TTV Species 3) |
| (F56): DLAEKTRMR | FR751488 (TTV Species 3) |
| (F57): NLAEAARNR | JN231329 (TTV Species 3) |
| (F58): TLCEQTRKK | KT163904 (TTV Species 3) |
| (F59): DAAKKIRTK | KT163917 (TTV Species 3) |
| (F60): QLAKITRER | MH648901 (TTV Species 3) |
| (F61): TLAEKTRAR | MH648958 (TTV Species 3) |

| | |
|---|---|
| (F62): DLTEITRNR | MH649092 (TTV Species 3) |
| (F63): SLCEKTRQK | MH649156 (TTV Species 3) |
| (F64): ELAEQTRQR | MH649242 (TTV Species 3) |
| (F65): ELAQKTRAR | MH649258 (TTV Species 3) |
| (F66): KLAKKTRER | MT501645 (TTV Species 3) |
| (F67): DLPEIVKKS | NC_014081 (TTV Species 3) |
| (F68): VTPGTTSSP | AF315076 (TTV Species 5)<br>AF345522 (TTV Species 5) |
| (F69): GNFKKNTEN | AJ620212 (TTV Species 6) |
| (F70): IWNQNTIQN | AJ620213 (TTV Species 6)<br>AJ620215 (TTV Species 6) |
| (F71): IWNPSTIQN | AJ620217 (TTV Species 6) |
| (F72): DSFKTNATN | AB054648 (TTV Species 7) |
| (F73): QIREKIKKN | AB064604 (TTV Species 9) |
| (F74): NIITKNMES | AB064606 (TTV Species 9) |
| (F75): AIIQKIVKN | AF345524 (TTV Species 9) |
| (F76): QVITENMES | AJ620227 (TTV Species 9)<br>AJ620228 (TTV Species 9)<br>AJ620229 (TTV Species 9)<br>AB064602 (TTV Species 29) |
| (F77): KILIKNMIT | AJ620230 (TTV Species 9)<br>AJ620231 (TTV Species 9)<br>AJ620233 (TTV Species 9)<br>AJ620235 (TTV Species 9) |
| (F78): NILIKNMIT | AJ620232 (TTV Species 9)<br>AJ620234 (TTV Species 9) |
| (F79): TIITKNMES | AY026465 (TTV Species 9) |
| (F80): QKIIKNMES | DQ186999 (TTV Species 9)<br>DQ187000 (TTV Species 9)<br>DQ187001 (TTV Species 9)<br>DQ187002 (TTV Species 9)<br>DQ187003 (TTV Species 9) |
| (F81): GILARNACT | DQ187004 (TTV Species 9)<br>DQ187005 (TTV Species 9) |
| (F82): QTAQTIRQT | NC_014075 (TTV Species 9) |
| (F83): TQNIKPDQN | GU797360 (TTV Species 10) |
| (F84): RDNNFALET | AF315077 (TTV Species 13)<br>AF345526 (TTV Species 13) |
| (F85): LTQLTATVP | AB060593 (TTV Species 15) |
| (F86): NPVSKKIWP | AF345521 (TTV Species 15) |
| (F87): QWYWTQLTS | AX025730 (TTV Species 15)<br>NC_014096 (TTV Species 15) |
| (F88): QWYWTKLTQ | AX025822 (TTV Species 15) |
| (F89): QHYWTQLTT | AY449524 (TTV Species 15) |
| (F90): YMLARRAWS | FR751498 (TTV Species 15)<br>FR751499 (TTV Species 15) |
| (F91): KIKKYRDQR | KM593802 (TTV Species 15) |

| | |
|---|---|
| (F92): NPVSKKVWP | NC_014091 (TTV Species 15) |
| (F93): EIPQTNVCH | AX025838 (TTV Species 18) |
| (F94): HVPITTTKP | AB060595 (TTV Species 20) |
| (F95): VPPTWTTPT | AF345527 (TTV Species 20) |
| (F96): TVQSVATSN | NC_038340 (TTV Species 20) |
| (F97): EEIGKAANE | AF371370 (TTV Species 24) |
| (F98): NIKSARSAY | AX174942 (TTV Species 24) |
| (F99): EIGSNANKW | DQ186994 (TTV Species 24)<br>DQ186995 (TTV Species 24) |
| (F100): LIQQEYMYH | AB038621 (TTV Species 29) |
| (F101): QKEYMYHYP | AB038624 (TTV Species 29) |
| (F102): TFSTHRSTR | AB064595 (TTV Species 29) |
| (F103): RINLDWMTP | AB064597 (TTV Species 29) |
| (F104): TRPKGKRSK | AB064598 (TTV Species 29) |
| (F105): KWIKIAEDG | AB064600 (TTV Species 29) |
| (F106): LIKAEYLYH | AB064603 (TTV Species 29) |

Figure S1

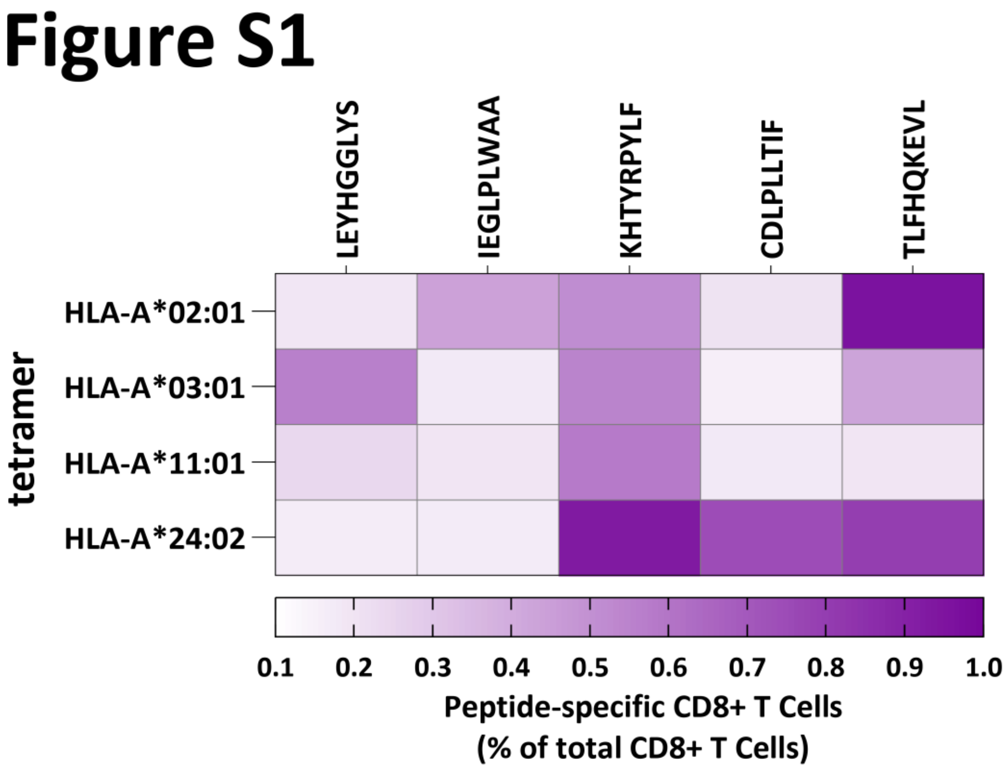


**Figure S1:** Torque Teno viruses elicit TTV-specific CD8$^+$ T cell responses. Total CD8+ T cells from 240 healthy blood donors (BD005-249) were pooled and stimulated with individual HLA-A*02:01, -A*03:01, -A*11:01, and -A*24:02 tetramers, loaded with individual TTV-derived LEYHGGLYS, IEGLPLWAA, KHTYRPYLF, CDLPLLTIF and TLFHQKEVL peptides. Heat map shows the frequency of TTV-peptide-specific CD8+ T cells.

Figure S2

## Figure S2A

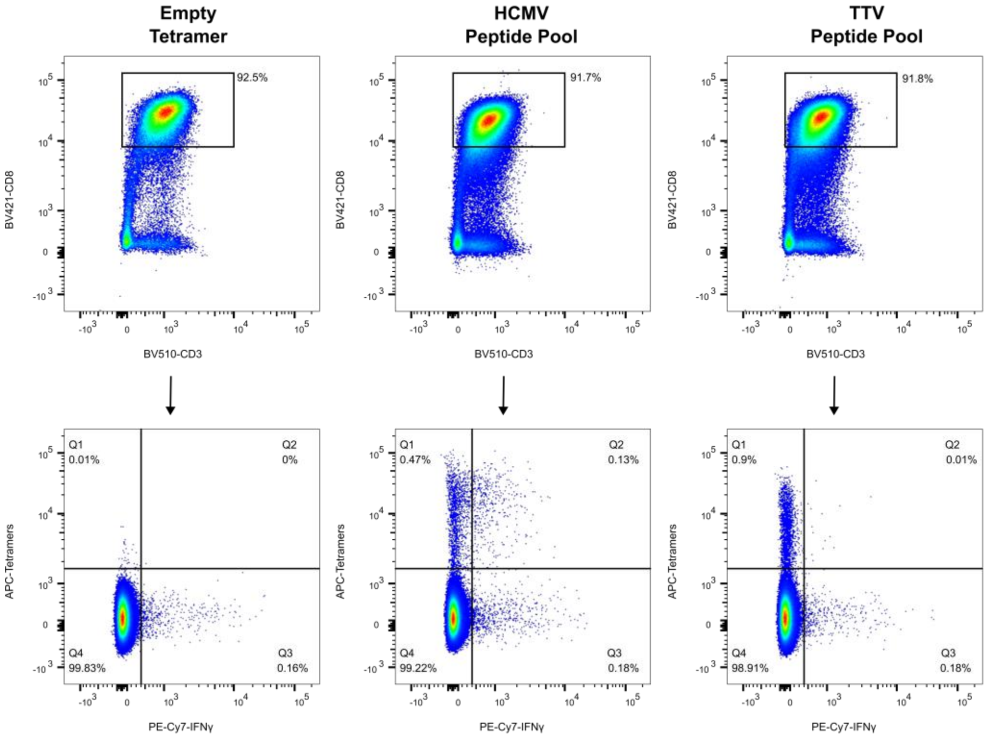


## Figure S2B

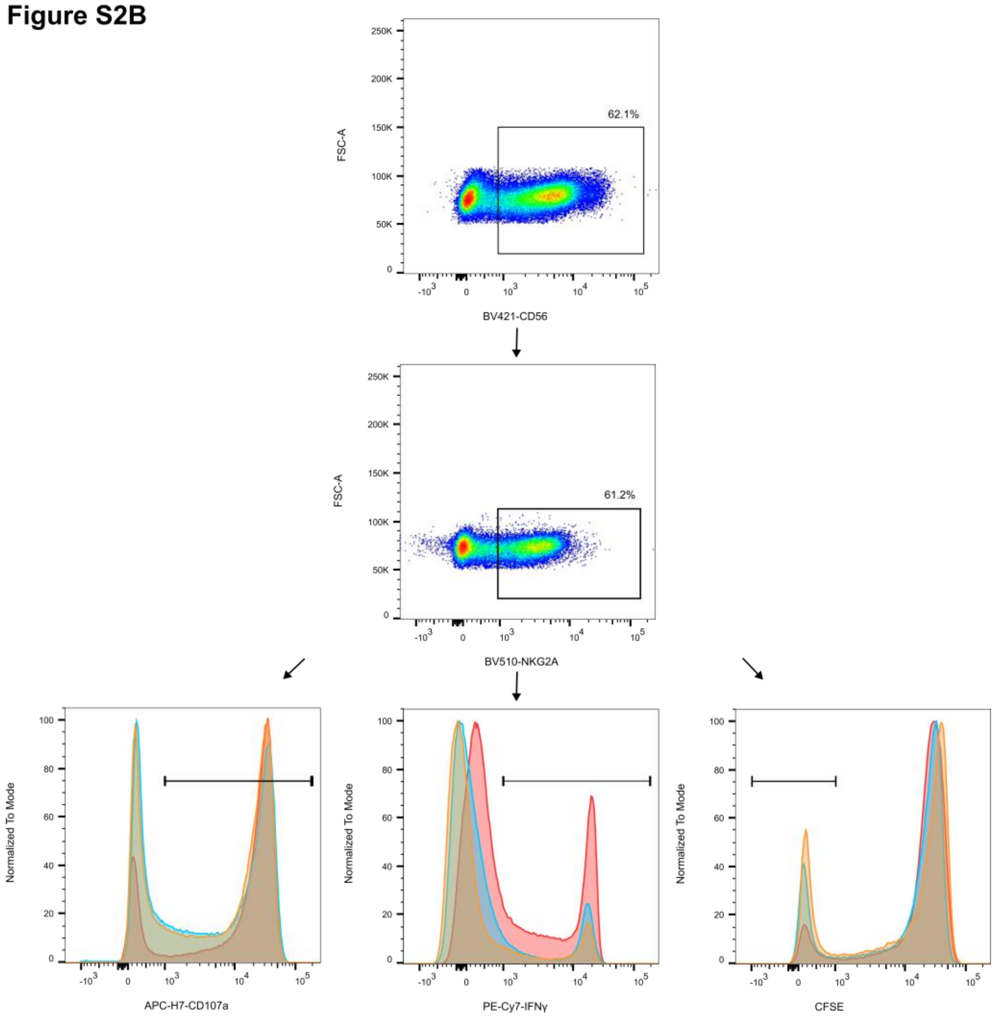

**Figure S2:** Gating Strategy: (A) Identification of activated HCMV-specific and TTV-specific $CD8^+$ T-cells. $CD8^+$ T-cells from one blood donor were cultured in the presence of APC-Tetramers alone (Empty Tetramer) or APC-Tetramers loaded with an HCMV-derived peptide pool (HCMV Peptide Pool) or a TTV-derived peptide pool (TTV Peptide Pool). Activated HCMV-specific and TTV-specific $CD8^+$ T-cells were identified as APC-Tetramers and PE-Cy7-IFNγ double positive cells (Q2). Gating strategy of one blood donor is shown. (B) $NKG2A^+$ inhibition and proliferation assay. K562-HLA-E*0103/0103 ($NKG2A^+$ inhibition assay) or RMA-S/HLA-E/LFA-3 cells ($NKG2A^+$ proliferation assay) were first incubated together with 300µM of the HCMV-encoded VMAPRTLIL (blue) or the TTV-encoded TTDKFTLRI (orange) or without peptides (red) and then incubated together with pre-activated and enriched $CD56^+$ NK-cells. Gating strategy for $NKG2A^+$ NK-cells of one blood donor is shown. $NKG2A^+$ inhibition assay: The percentage of (Left Panel) cytotoxic $CD107a^+NKG2A^+CD56^+$ NK-cells (VMAPRTLIL: 48.5%, TTDKFTLRI: 46.8%, without peptides: 60.9%) or (Middle Panel) $IFN\gamma^+NKG2A^+CD56^+$ NK-cells (VMAPRTLIL: 13.7%, TTDKFTLRI: 12.1%, without peptides: 25.8%) or (Right Panel) proliferating ($CFSE^{low}$) $NKG2A^+CD56^+$ NK-cells (VMAPRTLIL: 17.7%, TTDKFTLRI: 21.9%, without peptides) is shown.

Figure S3

# Figure S3

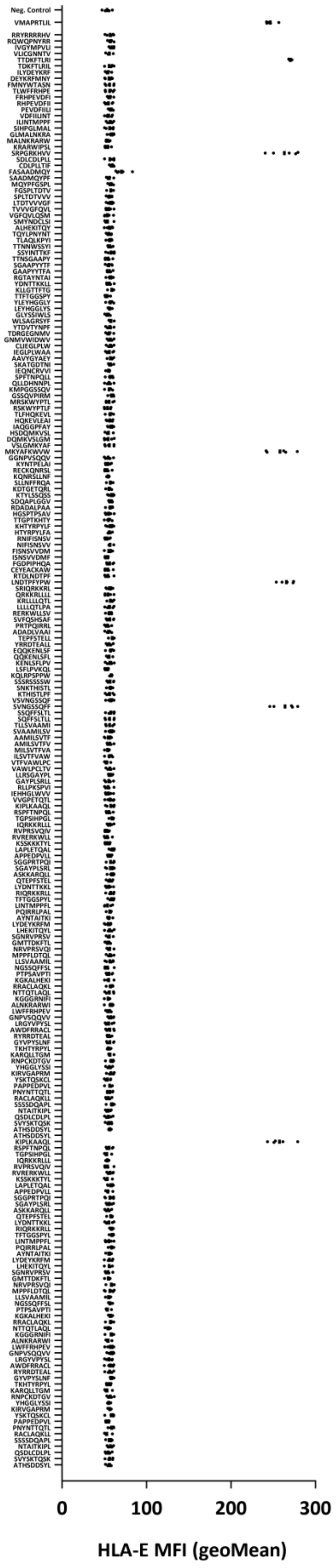

**Figure S3:** Distinct TTV-peptides upregulate HLA-E: HLA-E upregulation assay: RMA/S-HLA-E cells were incubated together with 300µM of the HCMV-encoded VMAPRTLIL or the indicated TTV-encoded peptides. The surface expression of HLA-E was then assessed by flow-cytometry. Each peptide was tested in five independent replicates.

Figure S4

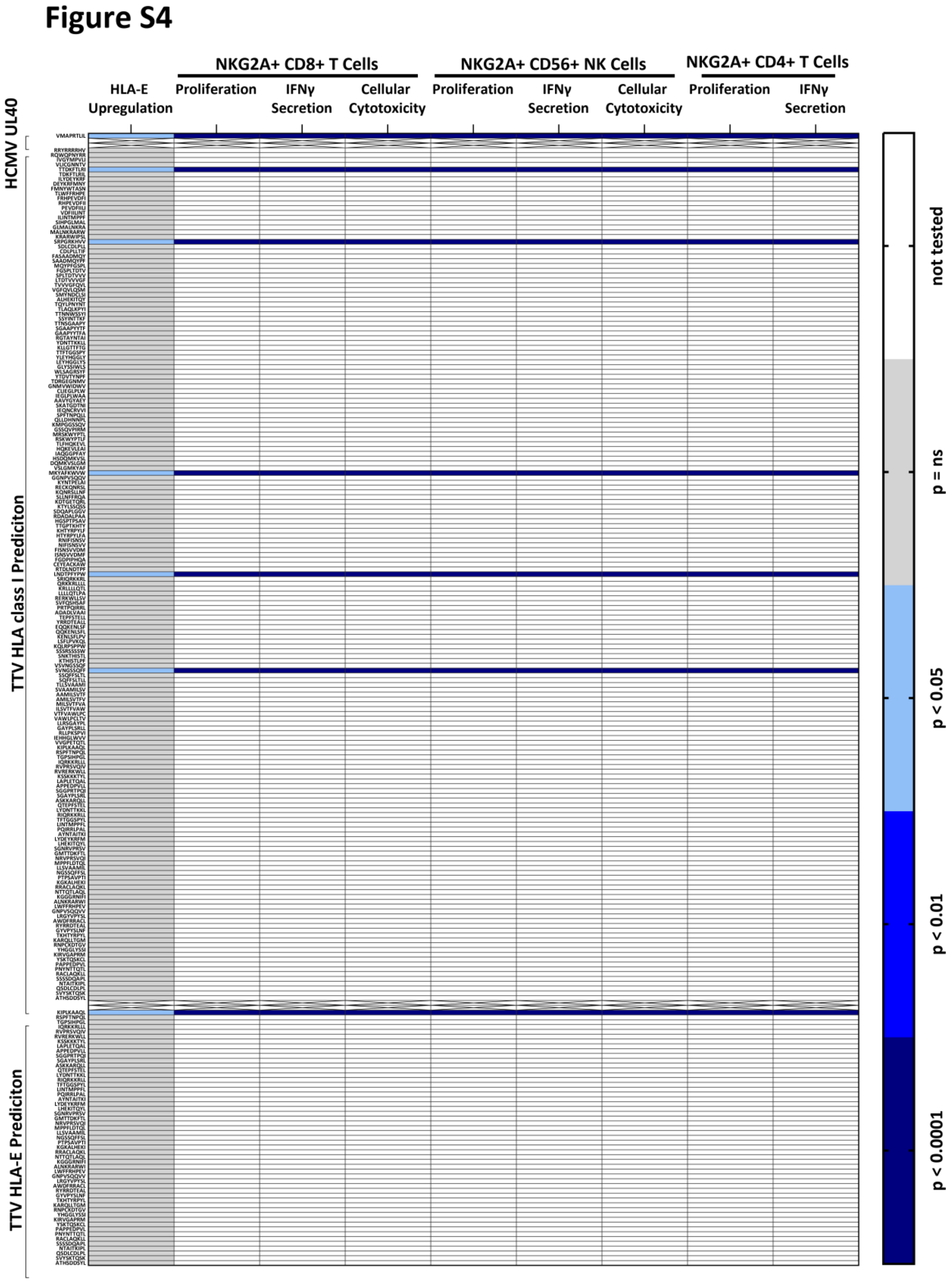
Figure S4
NKG2A+ CD8+ T Cells
NKG2A+ CD56+ NK Cells
NKG2A+ CD4+ T Cells
HLA-E Upregulation
Proliferation
IFNγ Secretion
Cellular Cytotoxicity
Proliferation
IFNγ Secretion
Cellular Cytotoxicity
Proliferation
IFNγ Secretion
HCMV UL40
TTV HLA class I Prediciton
TTV HLA-E Prediciton
not tested
p = ns
p < 0.05
p < 0.01
p < 0.0001

**Figure S4:** TTV-peptides elicit potent NKG2A$^+$-mediated effector functions: HLA-E upregulation assay: RMA/S-HLA-E cells were incubated together with 300µM of the HCMV-encoded VMAPRTLIL or the indicated TTV-encoded peptides. The surface expression of HLA-E was then assessed by flow-cytometry. NKG2A$^+$ inhibition and proliferation assay: K562-HLA-E*0103/0103 or RMA/S-HLA-E cells were first incubated together with 300µM of the HCMV-encoded or the indicated TTV-encoded peptide variants and then incubated together with pre-activated and enriched CD8$^+$ T-cells, CD56$^+$ NK-cells or CD4$^+$ T-cells. The percentage of cytotoxic CD107a$^+$NKG2A$^+$CD8$^+$ T-cells or CD107a$^+$NKG2A$^+$CD56$^+$ NK-cells as well as IFNγ$^+$NKG2A$^+$CD8$^+$ T-cells, IFNγ$^+$NKG2A$^+$CD4$^+$ T-cells or IFNγ$^+$NKG2A$^+$CD56$^+$ NK-cells as well as proliferating (CFSE$^{low}$) NKG2A$^+$CD8$^+$ T-cells, NKG2A$^+$CD4$^+$ T-cells or NKG2A$^+$CD56$^+$ NK-cells was assessed by flow-cytometry. Heat map shows the p-value in comparison to the non-peptide control of 240 independent biological replicates, reflecting N=120 healthy TTV-DNA-positive and HCMV-seropositive healthy blood donors (BD005-BD124) as well as 120 healthy TTV-DNA-positive and HCMV-seronegative healthy blood donors (BD125-BD249). **HCMV:** Human Cytomegalovirus, **IFNγ:** Interferon γ, **TTV:** Torque Teno Virus.

Figure S5

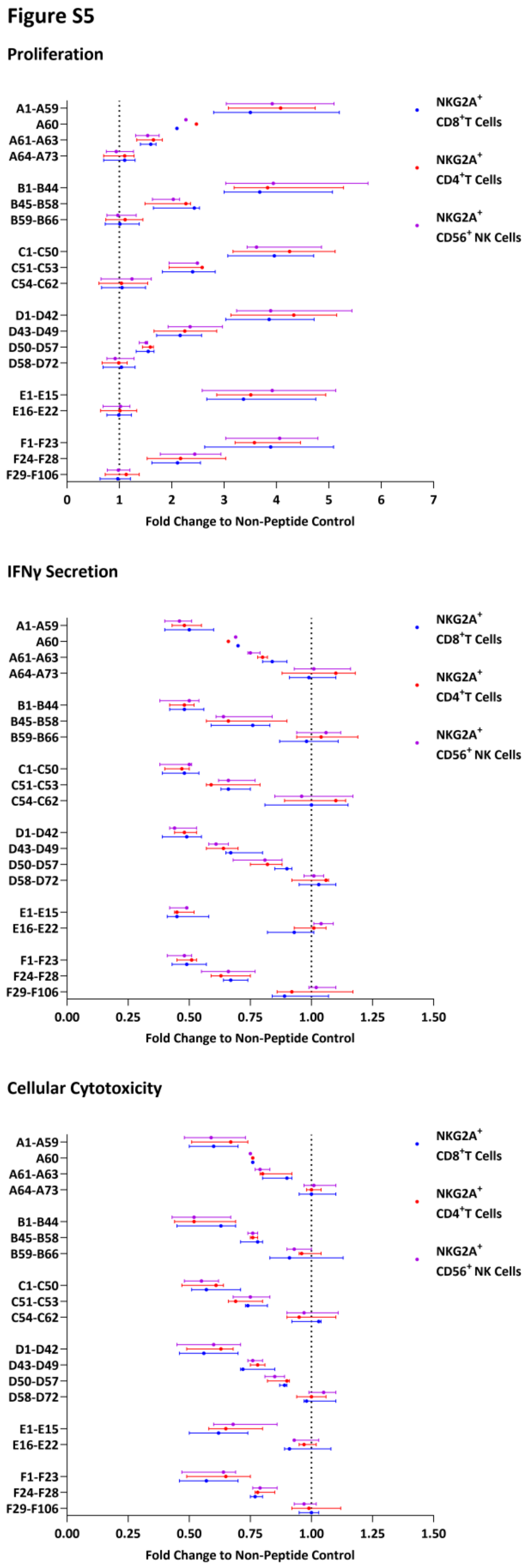
Figure S5
Proliferation
A1-A59
A60
A61-A63
A64-A73
B1-B44
B45-B58
B59-B66
C1-C50
C51-C53
C54-C62
D1-D42
D43-D49
D50-D57
D58-D72
E1-E15
E16-E22
F1-F23
F24-F28
F29-F106
0
1
2
3
4
5
6
7
Fold Change to Non-Peptide Control
NKG2A+ CD8+T Cells
NKG2A+ CD4+T Cells
NKG2A+ CD56+ NK Cells
IFNγ Secretion
0.00
0.25
0.50
0.75
1.00
1.25
1.50
Fold Change to Non-Peptide Control
Cellular Cytotoxicity
Fold Change to Non-Peptide Control

**Figure S5:** Distinct TTV-peptide variants elicit potent NKG2A$^+$-mediated effector functions: NKG2A$^+$ inhibition and proliferation assay: K562-HLA-E*0103/0103 or RMA/S-HLA-E cells were first incubated together with 300 µM of indicated TTV-encoded peptide variants (Table S2) and then incubated together with pre-activated and enriched CD8$^+$ T cells, CD56$^+$ NK cells or CD4$^+$ T cells. The percentage of cytotoxic CD107a$^+$NKG2A$^+$CD8$^+$ T cells or CD107a$^+$NKG2A$^+$CD56$^+$ NK cells as well as IFNγ$^+$NKG2A$^+$CD8$^+$ T cells, IFNγ$^+$NKG2A$^+$CD4$^+$ T cells or IFNγ$^+$NKG2A$^+$CD56$^+$ NK cells as well as proliferating (CFSE$^{low}$) NKG2A$^+$CD8$^+$ T cells, NKG2A$^+$CD4$^+$ T cells or NKG2A$^+$CD56$^+$ NK cells was assessed by flow-cytometry. The blots show the mean and min-max fold change of respective peptides in comparison to the non-peptide control of 240 independent biological replicates, reflecting N=120 healthy TTV-DNA-positive and HCMV-seropositive healthy blood donors (BD005-BD124) as well as 120 healthy TTV-DNA-positive and HCMV-seronegative healthy blood donors (BD125-BD249). Peptides were combined in respective groups according to the p-value in comparison to the non-peptide control of 240 independent biological replicates and numbed after NKG2A$^+$ inhibition and proliferation assays according to the statistical difference in comparison to the non-peptide control (Fig.3). **IFNγ:** Interferon γ.
